# Ligand Conversion in Nanocrystal Synthesis: The Oxidation of Alkylamines to Fatty Acids by Nitrate

**DOI:** 10.1021/jacsau.1c00349

**Published:** 2021-10-12

**Authors:** Mariano Calcabrini, Dietger Van den Eynden, Sergi Sánchez Ribot, Rohan Pokratath, Jordi Llorca, Jonathan De Roo, Maria Ibáñez

**Affiliations:** † IST Austria, Am Campus 1, 3400 Klosterneuburg, Austria; ‡ Department of Chemistry, University of Basel, 4058 Basel, Switzerland; § Institute of Energy Technologies, Department of Chemical Engineering and Barcelona Research Center in Multiscale Science and Engineering, Universitat Politecnica de Catalunya, 08019 Barcelona, Spain

**Keywords:** Oxides, Nanocrystals, Ceria, Nitrate, Oxidation, Ligand, NMR

## Abstract

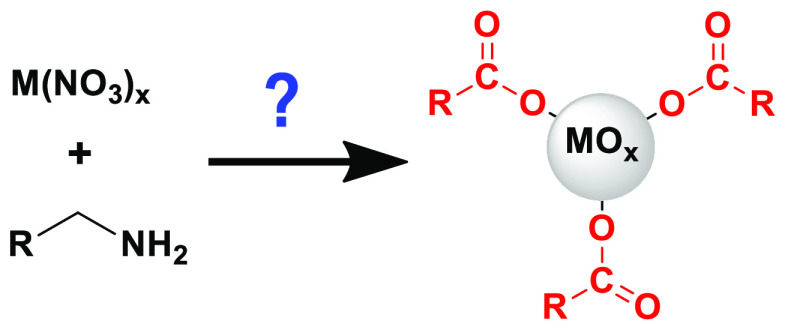

Ligands are a fundamental part of nanocrystals. They control and direct nanocrystal syntheses and provide colloidal stability. Bound ligands also affect the nanocrystals’ chemical reactivity and electronic structure. Surface chemistry is thus crucial to understand nanocrystal properties and functionality. Here, we investigate the synthesis of metal oxide nanocrystals (CeO_2-*x*_, ZnO, and NiO) from metal nitrate precursors, in the presence of oleylamine ligands. Surprisingly, the nanocrystals are capped exclusively with a fatty acid instead of oleylamine. Analysis of the reaction mixtures with nuclear magnetic resonance spectroscopy revealed several reaction byproducts and intermediates that are common to the decomposition of Ce, Zn, Ni, and Zr nitrate precursors. Our evidence supports the oxidation of alkylamine and formation of a carboxylic acid, thus unraveling this counterintuitive surface chemistry.

Ligand-assisted syntheses allow preparation of colloidally stable nanocrystals (NCs) with controlled size,^1−3^ shape,^4−7^ and composition.^8−10^ In these syntheses, long-chain aliphatic ligands are used to dissolve the precursors and control nucleation and growth.^11−13^ After the syntheses, some ligands remain bound to the NC surface providing colloidal stability and determining solubility,^14,15^ reactivity,^16−20^ and electronic structure.^21−25^ Therefore, unveiling the structure and binding motif of surface ligands is fundamental to understand NC properties,^26^ design ligand exchange strategies,^20,27−30^ and envision potential applications.^31,32^

In reactions where several organic ligands can bind to the NCs, the surface chemistry is typically studied in detail by nuclear magnetic resonance (NMR) spectroscopy, e.g., for CdSe,^28,33^ PbS,^34−36^ and InP^29,37,38^ NCs. However, in reactions with only one type of ligand, the NC surface is often assumed to be capped by this particular ligand. Previously, this assumption was proven wrong, with trioctylphosphine oxide decomposing to phosphinic and phosphonic acid ligands during the synthesis of several metal oxides.^39^

In the present work, we disclose an even more extreme example, where alkylamine ligands are oxidized into carboxylic acids during the synthesis of CeO_2-*x*_ NCs from cerium nitrate. NMR has proven to be a powerful tool to understand the reaction mechanisms in NC synthesis. For example, it has been used to explain the reduction of S with amines to prepare metal sulfide NCs,^40^ and to unveil the role of H_2_Se in the formation of CdSe NCs.^41,42^ Here, we use various NMR techniques to investigate the intermediates and reaction byproducts and propose a reaction path that we cross-examine with rigorous control experiments. We further analyzed the synthesis of other oxide NCs (NiO and ZnO) from the corresponding nitrates and found that the amine undergoes the same reactions. In a typical synthesis, Ce(NO_3_)_3_·6H_2_O is dissolved under vacuum in oleylamine and 1-octadecene forming a complex, [Ce(RNH_2_)_*n*_(NO_3_)_3_].^4,43^ Upon heating, this complex decomposes, yielding CeO_2-*x*_ NCs.
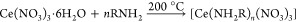

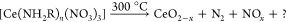


Herein, we use *n*-octadecane instead of 1-octadecene due to the latter’s tendency for spontaneous polymerization.^44^ In particular, cerium nitrate (1 mmol), oleylamine (6 mmol), and octadecane (4 mL) are degassed at room temperature and 80 °C for 30 min each. Under argon, the temperature is increased to 300 °C with a ramp of 15 °C per minute. The reaction mixture is kept at 300 °C for 60 min before cooling down. At 160 °C, during cooldown, 2 mL of toluene is injected. The as-synthesized NCs are precipitated with acetone (25 mL), and after centrifugation, the particles are resuspended in toluene (5 mL) and precipitated again with acetone (25 mL). This washing procedure is repeated twice, and the particles are finally stored in 5 mL toluene.

The purified NCs have a quasi-spherical shape, an average crystallite size of 6.5 nm, and a cubic crystallographic phase with space group *Fm*3̅*m* (Figures S1, S3, and S4).^4,45^ Using X-ray photoelectron spectroscopy (XPS), we determined that the particles have a composition CeO_1.74_ (Table S1). Furthermore, we performed thermogravimetric analysis of the NCs and found 15 wt % organics, corresponding to a ligand coverage of 3.3 ligands/nm^2^ (Figure S6), consistent with values reported for other oxide NCs.^14,46^

The broad resonances in the ^1^H NMR spectrum of the NCs indicate bound ligands (Figure 1).^47^ However, the broadening also prevents their identification. To overcome this limitation, we treat the NCs with trifluoroacetic acid, which strips the original ligands from the surface.^4,9^ Specifically, we add 10 μL of pure trifluoroacetic acid to a solution of 50 mg of NCs in 0.5 mL CDCl_3_. The particles precipitate, and the mixture is put in an ultrasonic bath for 30 min and subsequently dried under vacuum. CDCl_3_ (0.6 mL) are added, and after thorough mixing, the precipitate is filtered. The supernatant was measured in NMR. The stripped ligands exhibit sharp resonances consistent with the fingerprint of a fatty acid (Figure 1). Especially, the α resonance is diagnostic, aligning well with the α resonance of oleic acid (2.4 ppm) and clearly different from the α resonance of protonated oleylamine (2.7 ppm). The alkene resonance ε of the fatty acid reveals a mixture of cis and trans isomers, similar to that observed in commercial oleylamine,^48^ suggesting that the acid is formed from the amine (Figure S7). It should be noted that the integral of the alkene resonance is lower than expected, pointing to additional reactions concerning the double bond (Figure S8).

**Figure 1:**
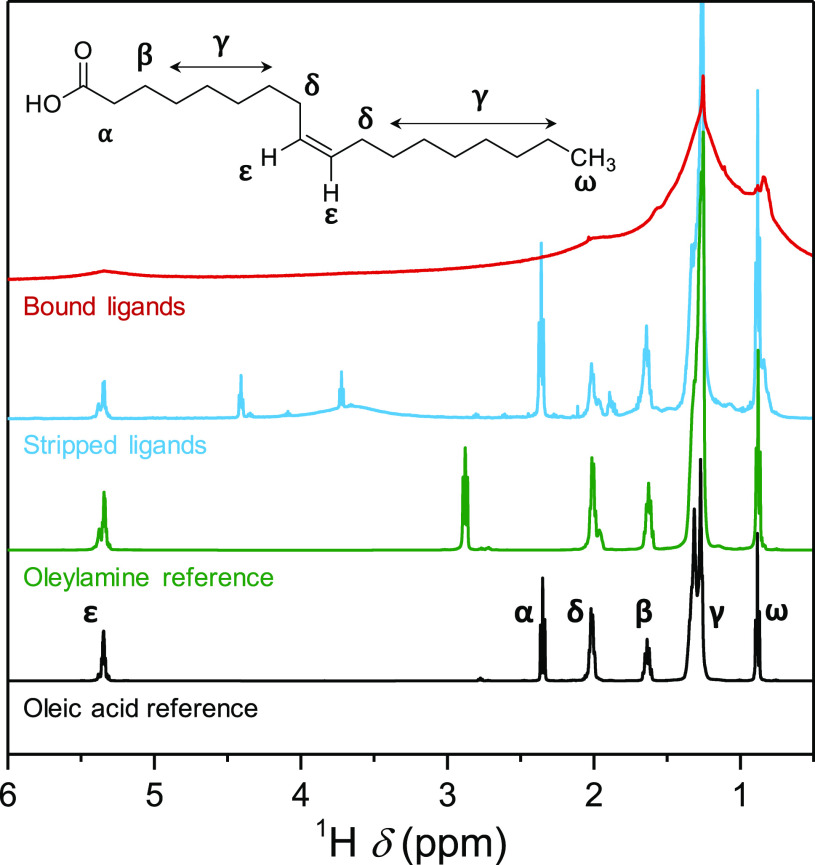
^1^H NMR spectra of purified NCs in benzene-*d*_6_, the stripped ligands (using trifluoroacetic acid) in CDCl_3_ and the oleylamine and oleic acid references in CDCl_3_ with additional trifluoroacetic acid added.

We set out to investigate the reaction path leading to the formation of the carboxylic acid. To avoid the reactivity of the double bond, we replaced oleylamine with hexadecylamine. This also simplifies the NMR spectra since hexadeylamine has no alkene resonance. The synthesis with hexadecylamine yields NCs with similar characteristics (size, shape, crystal structure; Figure S2). In addition, the NC surface is again capped by a fatty acid, in this case palmitic acid (hexadecanoic acid) (Figure S9).

Figure 2 shows the ^1^H NMR spectrum of the reaction mixture after completion of the synthesis. Besides the starting alkylamine and the solvent, 12 additional resonances are present in the ^1^H NMR spectrum, labeled *A* to *M* in Figure 2. Using advanced NMR spectroscopy, we assigned the resonances to six different compounds derived from hexadecylamine. The reaction mixture contains a secondary aldimine (**6**, resonances *A*, *G*, and *K*), a terminal alkene (**2**, resonances *B*, *E*, and *M*), an amide (**9**, resonances *D*, *H*, and *L*), an alcohol (**3**, resonance *F*), a nitrile (**5**, *J*), and yet unidentified compounds (resonances *C*, *I*). The identification of these compounds was further confirmed by adding either commercial or synthesized reference compounds to the reaction mixture (spiking experiments). The roadmap for the assignments, together with the complete data set, can be found in the SI (Figures S10–S22).

**Figure 2:**
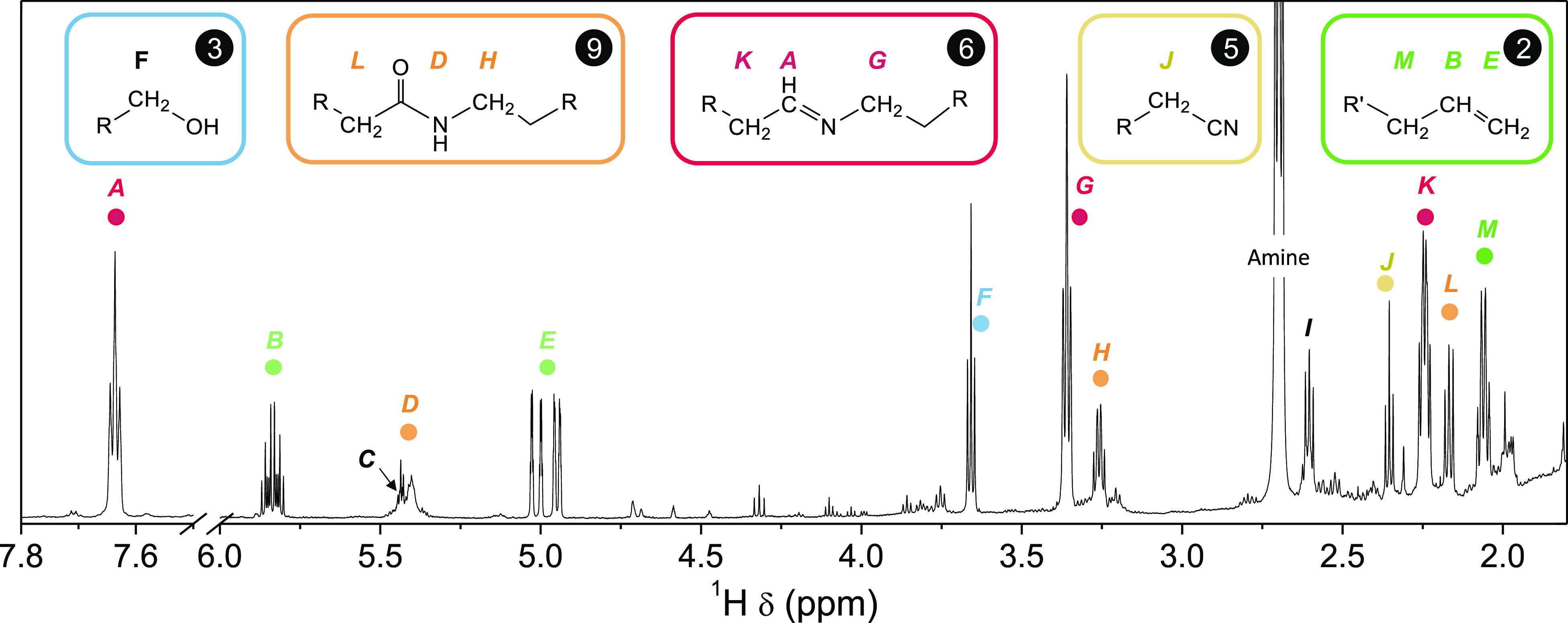
^1^H NMR spectrum of the reaction mixture of NCs prepared with hexadecylamine in octadecane after the completion of the synthesis. The most intense resonance, labeled “amine”, corresponds to hexadecylamine. Resonances *C* and *I* could not be assigned.

Scheme 1 illustrates our proposed reaction path from the alkylamine ligand into the different byproducts and intermediates. The different molecules are labeled **1**–**9** and are color-coded to indicate how these were identified. The coordinated amine **1**, referred to simply as the amine, reacts with nitrates in different ways. The alkene **2** and alcohol **3** are the expected products for the decomposition of alkylamines complexed to metal nitrates, as they have been reported for the decomposition of methylamine and ethylamine copper(II) nitrate complexes.^49^ The high reaction temperature could also promote the formation of the alkene **2** by β-elimination of the amine **1** under basic conditions.^50^

**Scheme 1:**
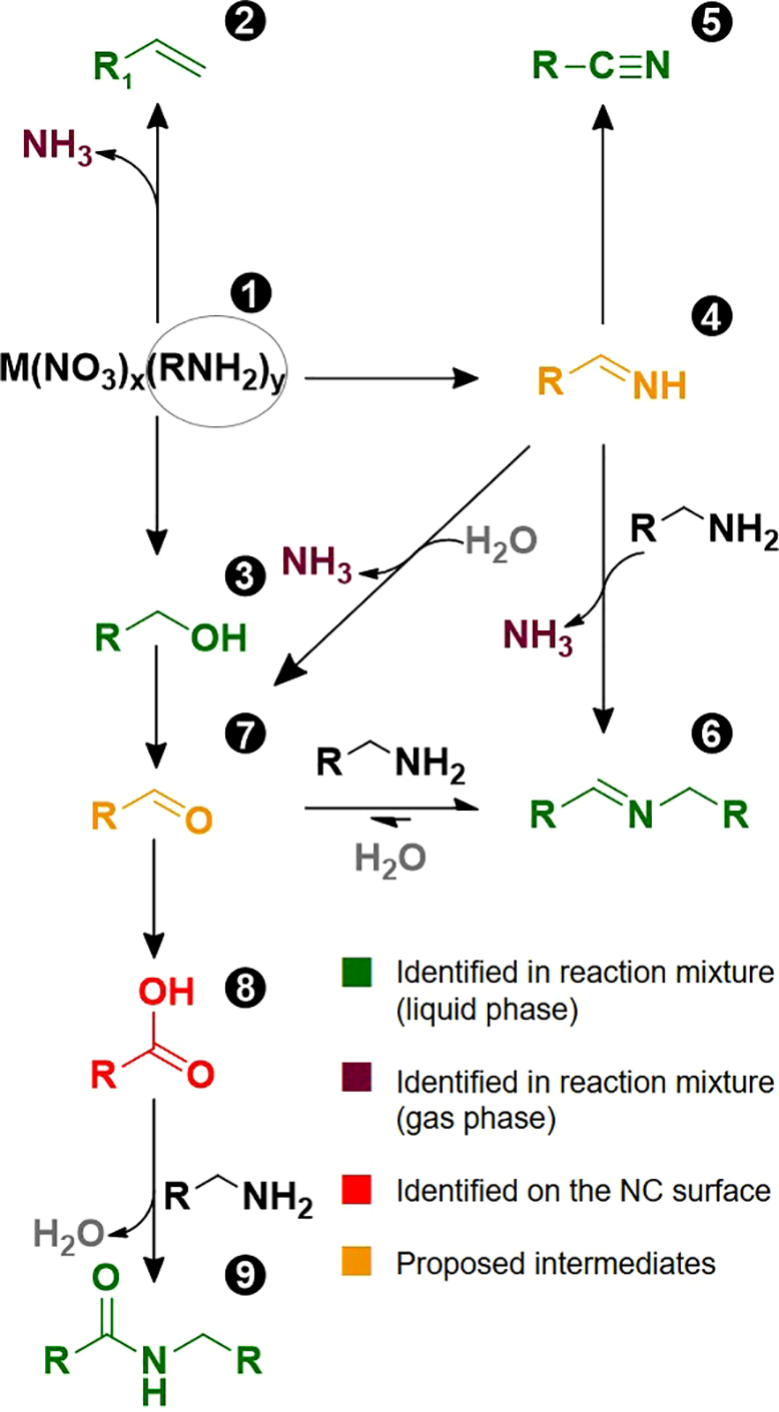
Proposed Reaction Path for the Formation of Carboxylic Acid through the Oxidation of Coordinated Alkylamines by Nitrate

The formation of the other identified byproducts involves the oxidation of the amine group. For the formation of the secondary aldimine **6** and the nitrile **5**, we proposed an intermediate: the primary aldimine **4**. We hypothesize that **1** is oxidized to **4** by nitrate. Then, **4** can be further oxidized to the nitrile **5**, which is observed in the reaction mixture.^51^ Furthermore, the primary aldimine **4** can condense with a second equivalent of amine **1** forming the more stable secondary aldimine **6**.^52,53^ Aldimine **6** is observed in the reaction mixture and is also a common byproduct of the synthesis of nitrides.^54^ The formation of **6** entails the evolution of ammonia, which we detected in the gas phase (see Supporting Information S16). The intermediate **4** could not detected in the reaction mixture (Figure 2), which we attribute to the low stability of primary aldimines.^55^ For the formation of the carboxylic acid **8** and the amide **9**, we propose aldehyde **7** as an intermediate. This aldehyde is the oxidation product of the alcohol **3**(49,56) or could be obtained by hydrolysis of either aldimine with adventitious water.^55^ The aldehyde **7** is further oxidized to the carboxylic acid **8**. Finally, the carboxylic acid **8** either binds to the NC surface or condenses with amine **1** to form amide **9**, explaining the absence of free carboxylic acid in the mixture.^57^ Aldehyde **7** was not detected in the mixture, but it appears as a logical intermediate. At the reaction temperature, it easily reacts with amine **1**, forming the secondary aldimine **6**. In control experiments with oleylamine and NaNO_3_ in octadecene the amine did not react, suggesting that the alkylamine needs to be coordinated to undergo these transformations (Figure S27).

Further insights into the chemical transformations were obtained by analyzing aliquots of the reaction mixture taken at different reaction temperatures during the heating process (Figures S23–S25). We analyzed the aliquots by ^1^H NMR and by transmission electron microscopy (TEM) to correlate the synthesis of the identified byproducts with the formation of the CeO_2-*x*_ NCs. We observed that at 100 °C the secondary aldimine **6** is already present, before any NCs and other bypodructs are formed (Figure S25). This observation indicates that the formation of the secondary aldimine precedes the oxidation of the amine to alcohol, aldehyde, and carboxylic acid. The amide **9**, the alkene **2**, and the alcohol **3** appear only in measurable concentrations above 200 °C when the complex starts decomposing, indicated by strong gas evolution and a darkening of the solution (Figure S23). These changes happen simultaneously with the formation of the NCs, as revealed by TEM (Figure S24). As the temperature increases to 300 °C, the NCs grow, but no new resonances appear in the ^1^H NMR spectrum of the reaction mixture (Figure S25).

To validate the proposed reaction path, we studied the synthesis of CeO_2-*x*_ NCs using a secondary amine. NCs are still formed with *N*,*N*-dioctadecylamine, and they have a quasispherical shape and similar size to those prepared with oleylamine (Figure S26). However, the second N-substituent on the ligand has two effects on the reaction path. First, the oxidation of the secondary amine immediately results in the stable, secondary aldimine **6**. Since the primmary aldimine **4** is not formed, oxidation to nitrile is prevented.

Second, *N,N*-dioctadecylamine has a more basic leaving group (p*K*_b_NH_3_ > p*K*_b_NH_2_R) and more steric hindrance than hexadecylamine. This severely hinders the formation of elimination and substitution products such as alkenes and alcohols. Indeed, we observe only a significant amount of secondary aldimine **6**, in the reaction mixture with *N,N*-dioctadecylamine (Figure S26, Table S2). Furthermore, the resulting NCs are capped by amine. To verify that the NCs synthesized with *N,N*-dioctadecylamine are acid-free, we studied the surface of the particles with XPS. Whereas the carboxylate features are clearly identified in the NCs synthesized with hexadecylamine, the ones synthesized with dioctadecylamine are acid free (Figure S5, Table S1). Despite the difference in surface chemistry, the change in the ligand used does not affect the stoichiometry, which was determined to be CeO_1.73_. The fact that we do not observe the fatty acid or the amide in the reaction mixture suggests that hydrolysis of the secondary aldimine **6**, is negligible under these conditions. Thus, the aldehyde **7** is most likely the direct oxidation product of the alcohol **3**, and the secondary aldimine does not react further to form the acid or amide.

Finally, to prove the generality of these results, we verified that the oxidation of amines by nitrate is not exclusive to the synthesis of CeO_2-*x*_ NCs. We decomposed Ni, Zn, and Zr nitrates in the presence of alkylamines and to form NiO and ZnO NCs, and colloidally unstable ZrO_2_ particles and found that in all cases, the composition of the reaction mixture is the same as for CeO_2-*x*_ and that, at least for ZnO NCs, the ligand shell is also composed only of carboxylic acid (Figure S27).

While there is precedent for alkylamines showing reactivity in NC synthesis (besides their function as ligand), the chemistry shown here is unique. Alkylamines have been used as reducing agents^40,58,59^ or as a source of ammonia.^54^ Certain side reactions were reported, such as the oxidation to nitriles,^51,60,61^ or the condensation with carboxylic acids.^57^ However, none of these previously reported transformations yielded byproducts with high binding affinity for the NC surface. In this work, we show that alkylamines are oxidized by nitrate to carboxylic acids, thus producing *in situ* another potential ligand. This transformation completely alters the final NC surface chemistry since the ligand shell is found only composed of carboxylate due to the higher binding affinity of carboxylates to metal oxide NCs, compared to amines.^27^

In summary, we scrutinized the synthesis of metal oxide NCs from metal nitrates in the presence of alkylamine ligands and revealed the oxidation of these amines with a comprehensive reaction scheme. We further proved that these reactions lead to the formation of carboxylic acids, which bind tightly to the NC surface, acting as the only capping ligand. Other NCs like metals and metal nitrides are also often synthesized from metal nitrates. Therefore, our current results might be relevant to understand these systems too.

## Supporting Information Available

Chemicals used, synthesis protocols, NMR experiments supporting the identification of the compounds (^1^H NMR, ^13^C NMR, COSY, HMBC, and HSQC spectra, and reference-spiking experiments) XRD, TEM, XPS measurements, and PDF analysis (PDF)

## References

[ref1] Gomes R.; Hassinen A.; Szczygiel A.; Zhao Q.; Vantomme A.; Martins J. C.; Hens Z. Binding of Phosphonic Acids to CdSe Quantum Dots: A Solution NMR Study. J. Phys. Chem. Lett. 2011, 2 (3), 145–152. 10.1021/jz1016729.

[ref2] Jansons A. W.; Hutchison J. E. Continuous Growth of Metal Oxide Nanocrystals: Enhanced Control of Nanocrystal Size and Radial Dopant Distribution. ACS Nano 2016, 10 (7), 6942–6951. 10.1021/acsnano.6b02796. 27328328

[ref3] Geisenhoff J. Q.; Tamura A. K.; Schimpf A. M. Using Ligands to Control Reactivity, Size and Phase in the Colloidal Synthesis of WSe_2_ Nanocrystals. Chem. Commun. 2019, 55 (60), 8856–8859. 10.1039/C9CC03326B. 31204745

[ref4] Berestok T.; Guardia P.; Blanco J.; Nafria R.; Torruella P.; López-Conesa L.; Estradé S.; Ibáñez M.; De Roo J.; Luo Z.; Cadavid D.; Martins J. C.; Kovalenko M. V.; Peiró F.; Cabot A. Tuning Branching in Ceria Nanocrystals. Chem. Mater. 2017, 29 (10), 4418–4424. 10.1021/acs.chemmater.7b00896.

[ref5] An K.; Kwon S. G.; Park M.; Na H. B.; Baik S.-I.; Yu J. H.; Kim D.; Son J. S.; Kim Y. W.; Song I. C.; Moon W. K.; Park H. M.; Hyeon T. Synthesis of Uniform Hollow Oxide Nanoparticles through Nanoscale Acid Etching. Nano Lett. 2008, 8 (12), 4252–4258. 10.1021/nl8019467. 19367964

[ref6] Kwon S. G.; Hyeon T. Colloidal Chemical Synthesis and Formation Kinetics of Uniformly Sized Nanocrystals of Metals, Oxides, and Chalcogenides. Acc. Chem. Res. 2008, 41 (12), 1696–1709. 10.1021/ar8000537. 18681462

[ref7] Kim K.; Reimnitz L. C.; Cho S. H.; Noh J.; Dong Z.; Gibbs S. L.; Korgel B. A.; Milliron D. J. Effect of Nonincorporative Cations on the Size and Shape of Indium Oxide Nanocrystals. Chem. Mater. 2020, 32 (21), 9347–9354. 10.1021/acs.chemmater.0c03281.

[ref8] Sytnyk M.; Kirchschlager R.; Bodnarchuk M. I.; Primetzhofer D.; Kriegner D.; Enser H.; Stangl J.; Bauer P.; Voith M.; Hassel A. W.; Krumeich F.; Ludwig F.; Meingast A.; Kothleitner G.; Kovalenko M. V.; Heiss W. Tuning the Magnetic Properties of Metal Oxide Nanocrystal Heterostructures by Cation Exchange. Nano Lett. 2013, 13 (2), 586–593. 10.1021/nl304115r. 23362940 PMC3573734

[ref9] Liu Y.; Cadavid D.; Ibáñez M.; De Roo J.; Ortega S.; Dobrozhan O.; Kovalenko M.; Cabot A. Colloidal AgSbSe_2_ Nanocrystals: Surface Analysis, Electronic Doping and Processing into Thermoelectric Nanomaterials. J. Mater. Chem. C 2016, 4 (21), 4756–4762. 10.1039/C6TC00893C.

[ref10] Yarema O.; Yarema M.; Wood V. Tuning the Composition of Multicomponent Semiconductor Nanocrystals: The Case of I-III-VI Materials. Chem. Mater. 2018, 30 (5), 1446–1461. 10.1021/acs.chemmater.7b04710.

[ref11] Yin Y.; Alivisatos A. P. Colloidal Nanocrystal Synthesis and the Organic-Inorganic Interface. Nature 2005, 437 (7059), 664–670. 10.1038/nature04165. 16193041

[ref12] Heuer-Jungemann A.; Feliu N.; Bakaimi I.; Hamaly M.; Alkilany A.; Chakraborty I.; Masood A.; Casula M. F.; Kostopoulou A.; Oh E.; Susumu K.; Stewart M. H.; Medintz I. L.; Stratakis E.; Parak W. J.; Kanaras A. G. The Role of Ligands in the Chemical Synthesis and Applications of Inorganic Nanoparticles. Chem. Rev. 2019, 119 (8), 4819–4880. 10.1021/acs.chemrev.8b00733. 30920815

[ref13] van Embden J.; Chesman A. S. R.; Jasieniak J. J. The Heat-Up Synthesis of Colloidal Nanocrystals. Chem. Mater. 2015, 27 (7), 2246–2285. 10.1021/cm5028964.

[ref14] De Roo J.; Van Den Broeck F.; De Keukeleere K.; Martins J. C.; Van Driessche I.; Hens Z. Unravelling the Surface Chemistry of Metal Oxide Nanocrystals, the Role of Acids and Bases. J. Am. Chem. Soc. 2014, 136 (27), 9650–9657. 10.1021/ja5032979. 24945901

[ref15] Yang Y.; Qin H.; Jiang M.; Lin L.; Fu T.; Dai X.; Zhang Z.; Niu Y.; Cao H.; Jin Y.; Zhao F.; Peng X. Entropic Ligands for Nanocrystals: From Unexpected Solution Properties to Outstanding Processability. Nano Lett. 2016, 16 (4), 2133–2138. 10.1021/acs.nanolett.6b00730. 26923682

[ref16] Knowles K. E.; Tagliazucchi M.; Malicki M.; Swenson N. K.; Weiss E. A. Electron Transfer as a Probe of the Permeability of Organic Monolayers on the Surfaces of Colloidal PbS Quantum Dots. J. Phys. Chem. C 2013, 117 (30), 15849–15857. 10.1021/jp406485y.

[ref17] Shi M.; Kwon H. S.; Peng Z.; Elder A.; Yang H. Effects of Surface Chemistry on the Generation of Reactive Oxygen Species by Copper Nanoparticles. ACS Nano 2012, 6 (3), 2157–2164. 10.1021/nn300445d. 22390268 PMC3314088

[ref18] Gabka G.; Bujak P.; Gryszel M.; Kotwica K.; Pron A. Anchor Groups Effect on Spectroscopic and Electrochemical Properties of Quaternary Nanocrystals Cu-In-Zn-S Capped with Arylamine Derivatives. J. Phys. Chem. C 2015, 119 (17), 9656–9664. 10.1021/acs.jpcc.5b02402.

[ref19] Irtem E.; Arenas Esteban D.; Duarte M.; Choukroun D.; Lee S.; Ibáñez M.; Bals S.; Breugelmans T. Ligand-Mode Directed Selectivity in Cu-Ag Core-Shell Based Gas Diffusion Electrodes for CO_2_ Electroreduction. ACS Catal. 2020, 10 (22), 13468–13478. 10.1021/acscatal.0c03210.

[ref20] Elimelech O.; Aviv O.; Oded M.; Banin U. A Tale of Tails: Thermodynamics of CdSe Nanocrystal Surface Ligand Exchange. Nano Lett. 2020, 20 (9), 6396–6403. 10.1021/acs.nanolett.0c01913. 32787157

[ref21] Kroupa D. M.; Vörös M.; Brawand N. P.; McNichols B. W.; Miller E. M.; Gu J.; Nozik A. J.; Sellinger A.; Galli G.; Beard M. C. Tuning Colloidal Quantum Dot Band Edge Positions through Solution-Phase Surface Chemistry Modification. Nat. Commun. 2017, 8 (1), 1–8. 10.1038/ncomms15257. 28508866 PMC5440806

[ref22] Anderson N. C.; Owen J. S. Soluble, Chloride-Terminated CdSe Nanocrystals: Ligand Exchange Monitored by ^1^H and ^31^P NMR Spectroscopy. Chem. Mater. 2013, 25 (1), 69–76. 10.1021/cm303219a.

[ref23] Wei J.; Schaeffer N.; Pileni M. P. Ag Nanocrystals: 1. Effect of Ligands on Plasmonic Properties. J. Phys. Chem. B 2014, 118 (49), 14070–14075. 10.1021/jp5050699. 25198062

[ref24] Ibáñez M.; Hasler R.; Genç A.; Liu Y.; Kuster B.; Schuster M.; Dobrozhan O.; Cadavid D.; Arbiol J.; Cabot A.; Kovalenko M. V. Ligand-Mediated Band Engineering in Bottom-up Assembled SnTe Nanocomposites for Thermoelectric Energy Conversion. J. Am. Chem. Soc. 2019, 141 (20), 8025–8029. 10.1021/jacs.9b01394. 31017419 PMC6588270

[ref25] Vale B. R. C.; Mourão R. S.; Bettini J.; Sousa J. C. L.; Ferrari J. L.; Reiss P.; Aldakov D.; Schiavon M. A. Ligand Induced Switching of the Band Alignment in Aqueous Synthesized CdTe/CdS Core/Shell Nanocrystals. Sci. Rep. 2019, 9 (1), 1–12. 10.1038/s41598-019-44787-y. 31171820 PMC6554334

[ref26] Boles M. A.; Ling D.; Hyeon T.; Talapin D. V. The Surface Science of Nanocrystals. Nat. Mater. 2016, 15 (2), 141–153. 10.1038/nmat4526. 26796733

[ref27] De Roo J.; Justo Y.; De Keukeleere K.; Van Den Broeck F.; Martins J. C.; Van Driessche I.; Hens Z. Carboxylic-Acid-Passivated Metal Oxide Nanocrystals: Ligand Exchange Characteristics of a New Binding Motif. Angew. Chem., Int. Ed. 2015, 54 (22), 6488–6491. 10.1002/anie.201500965. 25866095

[ref28] Drijvers E.; De Roo J.; Martins J. C.; Infante I.; Hens Z. Ligand Displacement Exposes Binding Site Heterogeneity on CdSe Nanocrystal Surfaces. Chem. Mater. 2018, 30 (3), 1178–1186. 10.1021/acs.chemmater.7b05362.

[ref29] Calvin J. J.; O’Brien E. A.; Sedlak A. B.; Balan A. D.; Alivisatos A. P. Thermodynamics of Composition Dependent Ligand Exchange on the Surfaces of Colloidal Indium Phosphide Quantum Dots. ACS Nano 2021, 15 (1), 1407–1420. 10.1021/acsnano.0c08683. 33404231

[ref30] Ibáñez M.; Korkosz R. J.; Luo Z.; Riba P.; Cadavid D.; Ortega S.; Cabot A.; Kanatzidis M. G. Electron Doping in Bottom-up Engineered Thermoelectric Nanomaterials through HCl-Mediated Ligand Displacement. J. Am. Chem. Soc. 2015, 137 (12), 4046–4049. 10.1021/jacs.5b00091. 25762361

[ref31] Liu Y.; Gibbs M.; Puthussery J.; Gaik S.; Ihly R.; Hillhouse H. W.; Law M. Dependence of Carrier Mobility on Nanocrystal Size and Ligand Length in PbSe Nanocrystal Solids. Nano Lett. 2010, 10 (5), 1960–1969. 10.1021/nl101284k. 20405957

[ref32] Ibáñez M.; Genc A.; Hasler R.; Liu Y.; Dobrozhan O.; Nazarenko O.; de la Mata M.; Arbiol J.; Cabot A.; Kovalenko M. V. Tuning Transport Properties in Thermoelectric Nanocomposites through Inorganic Ligands and Heterostructured Building Blocks. ACS Nano 2019, 13 (6), 6572–6580. 10.1021/acsnano.9b00346. 31185159 PMC6595432

[ref33] Zeng B.; Palui G.; Zhang C.; Zhan N.; Wang W.; Ji X.; Chen B.; Mattoussi H. Characterization of the Ligand Capping of Hydrophobic CdSe-ZnS Quantum Dots Using NMR Spectroscopy. Chem. Mater. 2018, 30 (1), 225–238. 10.1021/acs.chemmater.7b04204.

[ref34] Boles M. A.; Talapin D. V. Binary Assembly of PbS and Au Nanocrystals: Patchy PbS Surface Ligand Coverage Stabilizes the CuAu Superlattice. ACS Nano 2019, 13 (5), 5375–5384. 10.1021/acsnano.9b00006. 31017762

[ref35] Moreels I.; Justo Y.; De Geyter B.; Haustraete K.; Martins J. C.; Hens Z. Size-Tunable, Bright, and Stable PbS Quantum Dots: A Surface Chemistry Study. ACS Nano 2011, 5 (3), 2004–2012. 10.1021/nn103050w. 21355621

[ref36] Kessler M. L.; Dempsey J. L. Mapping the Topology of PbS Nanocrystals through Displacement Isotherms of Surface-Bound Metal Oleate Complexes. Chem. Mater. 2020, 32, 2561 10.1021/acs.chemmater.0c00014.

[ref37] Leemans J.; Dümbgen K. C.; Minjauw M. M.; Zhao Q.; Vantomme A.; Infante I.; Detavernier C.; Hens Z. Acid-Base Mediated Ligand Exchange on Near-Infrared Absorbing, Indium-Based III-V Colloidal Quantum Dots. J. Am. Chem. Soc. 2021, 143 (11), 4290–4301. 10.1021/jacs.0c12871. 33710882

[ref38] Hanrahan M. P.; Stein J. L.; Park N.; Cossairt B. M.; Rossini A. J. Elucidating the Location of Cd^2+^ in Post-Synthetically Treated InP Quantum Dots Using Dynamic Nuclear Polarization ^31^P and ^113^Cd Solid-State NMR Spectroscopy. J. Phys. Chem. C 2021, 125 (5), 2956–2965. 10.1021/acs.jpcc.0c09601.

[ref39] De Keukeleere K.; Coucke S.; De Canck E.; Van Der Voort P.; Delpech F.; Coppel Y.; Hens Z.; Van Driessche I.; Owen J. S.; De Roo J. Stabilization of Colloidal Ti, Zr, and Hf Oxide Nanocrystals by Protonated Tri-n-Octylphosphine Oxide (TOPO) and Its Decomposition Products. Chem. Mater. 2017, 29 (23), 10233–10242. 10.1021/acs.chemmater.7b04580.

[ref40] Thomson J. W.; Nagashima K.; Macdonald P. M.; Ozin G. A. From Sulfur–Amine Solutions to Metal Sulfide Nanocrystals: Peering into the Oleylamine–Sulfur Black Box. J. Am. Chem. Soc. 2011, 133 (13), 5036–5041. 10.1021/ja1109997. 21384888

[ref41] Frenette L. C.; Krauss T. D. Uncovering Active Precursors in Colloidal Quantum Dot Synthesis. Nat. Commun. 2017, 8 ( (1), ). 10.1038/s41467-017-01936-z PMC5727186 29233976

[ref42] Liu H.; Owen J. S.; Alivisatos A. P. Mechanistic Study of Precursor Evolution in Colloidal Group II-VI Semiconductor Nanocrystal Synthesis. J. Am. Chem. Soc. 2007, 129 (2), 305–312. 10.1021/ja0656696. 17212409

[ref43] Sridaeng D.; Limsirinawa A.; Sirojpornphasut P.; Chawiwannakorn S.; Chantarasiri N. Metal Acetylacetonate-Amine and Metal Nitrate-Amine Complexes as Low-Emission Catalysts for Rigid Polyurethane Foam Preparation. J. Appl. Polym. Sci. 2015, 132 (31), 42332 10.1002/app.42332.

[ref44] Dhaene E.; Billet J.; Bennett E.; Van Driessche I.; De Roo J. The Trouble with ODE: Polymerization during Nanocrystal Synthesis. Nano Lett. 2019, 19 (10), 7411–7417. 10.1021/acs.nanolett.9b03088. 31525055

[ref45] Yu T.; Joo J.; Park Y. Il; Hyeon T. Large-Scale Nonhydrolytic Sol-Gel Synthesis of Uniform-Sized Ceria Nanocrystals with Spherical, Wire, and Tadpole Shapes. Angew. Chem., Int. Ed. 2005, 44 (45), 7411–7414. 10.1002/anie.200500992. 16247811

[ref46] Valdez C. N.; Schimpf A. M.; Gamelin D. R.; Mayer J. M. Low Capping Group Surface Density on Zinc Oxide Nanocrystals. ACS Nano 2014, 8 (9), 9463–9470. 10.1021/nn503603e. 25131410

[ref47] De Roo J.; Yazdani N.; Drijvers E.; Lauria A.; Maes J.; Owen J. S.; Van Driessche I.; Niederberger M.; Wood V.; Martins J. C.; Infante I.; Hens Z. Probing Solvent-Ligand Interactions in Colloidal Nanocrystals by the NMR Line Broadening. Chem. Mater. 2018, 30 (15), 5485–5492. 10.1021/acs.chemmater.8b02523.

[ref48] Baranov D.; Lynch M. J.; Curtis A. C.; Carollo A. R.; Douglass C. R.; Mateo-Tejada A. M.; Jonas D. M. Purification of Oleylamine for Materials Synthesis and Spectroscopic Diagnostics for Trans Isomers. Chem. Mater. 2019, 31 (4), 1223–1230. 10.1021/acs.chemmater.8b04198.

[ref49] Southern T. M.; Wendlandt W. W. The Thermal Decomposition of Metal Complexes-XX. Some Amine Copper(II) Nitrate Complexes. J. Inorg. Nucl. Chem. 1970, 32 (12), 3783–3792. 10.1016/0022-1902(70)80552-8.

[ref50] Moldoveanu S. C. Pyrolysis of Amines and Imines. In Techniques and Instrumentation in Analytical Chemistry; Moldoveanu S. C., Ed.; Elsevier, 2010; Vol. 28, pp 349–364.

[ref51] Hiramatsu H.; Osterloh F. E. A Simple Large-Scale Synthesis of Nearly Monodisperse Gold and Silver Nanoparticles with Adjustable Sizes and with Exchangeable Surfactants. Chem. Mater. 2004, 16 (13), 2509–2511. 10.1021/cm049532v.

[ref52] Kobayashi S.; Nagayama S. Aldehydes vs Aldimines. Unprecedented Reactivity in Their Enolate Addition Reactions. J. Org. Chem. 1997, 62 (2), 232–233. 10.1021/jo962010h. 11671389

[ref53] Clayden J.; Greeves N.; Warren S. Organic Chemistry, 2nd ed.; Oxford, New York, 2012.

[ref54] Chen Y.; Landes N. T.; Little D. J.; Beaulac R. Conversion Mechanism of Soluble Alkylamide Precursors for the Synthesis of Colloidal Nitride Nanomaterials. J. Am. Chem. Soc. 2018, 140 (33), 10421–10424. 10.1021/jacs.8b06063. 30081636

[ref55] Layer R. W. The Chemistry of Imines. Chem. Rev. 1963, 63 (5), 489–510. 10.1021/cr60225a003.

[ref56] Rawalay S. S.; Shechter H. Oxidation of Primary, Secondary, and Tertiary Amines with Neutral Permanganate. A Simple Method for Degrading Amines to Aldehydes and Ketones. J. Org. Chem. 1967, 32 (10), 3129–3131. 10.1021/jo01285a042.

[ref57] De Roo J.; Van Driessche I.; Martins J. C.; Hens Z. Colloidal Metal Oxide Nanocrystal Catalysis by Sustained Chemically Driven Ligand Displacement. Nat. Mater. 2016, 15 (5), 517–521. 10.1038/nmat4554. 26808460

[ref58] Mourdikoudis S.; Liz-Marzán L. M. Oleylamine in Nanoparticle Synthesis. Chem. Mater. 2013, 25 (9), 1465–1476. 10.1021/cm4000476.

[ref59] Li Z.; Ji Y.; Xie R.; Grisham S. Y.; Peng X. Correlation of CdS Nanocrystal Formation with Elemental Sulfur Activation and Its Implication in Synthetic Development. J. Am. Chem. Soc. 2011, 133 (43), 17248–17256. 10.1021/ja204538f. 21939230

[ref60] Kadzutu-Sithole R.; Machogo-Phao L. F. E.; Kolokoto T.; Zimuwandeyi M.; Gqoba S. S.; Mubiayi K. P.; Moloto M. J.; Van Wyk J.; Moloto N. Elucidating the Effect of Precursor Decomposition Time on the Structural and Optical Properties of Copper (I) Nitride Nanocubes. RSC Adv. 2020, 10 (56), 34231–34246. 10.1039/C9RA09546B. PMC9056776 35519021

[ref61] Chen M.; Feng Y. G.; Wang X.; Li T. C.; Zhang J. Y.; Qian D. J. Silver Nanoparticles Capped by Oleylamine: Formation, Growth, and Self-Organization. Langmuir 2007, 23 (10), 5296–5304. 10.1021/la700553d. 17425348

