# Supplementary material for: Ligand Conversion in Nanocrystal Synthesis: The Oxidation of Alkylamines to Fatty Acids by Nitrate
Source: JACS Au. 2021 Oct 12;1(11):1898–903. doi: 10.1021/jacsau.1c00349 (PMC8611721; doi:10.1021/jacsau.1c00349)
Supplement: Supplementary file 1 — au1c00349_si_001.pdf [file au1c00349_si_001.pdf]

Supporting Information

Ligand Conversion in Nanocrystal Synthesis: The Oxidation of Alkylamines to Fatty Acids by Nitrate

Mariano Calcabrini§, Dietger Van den Eynden†, Sergi Sánchez Ribot§, Rohan Pokratath†, Jordi Llorca‡, Jonathan De Roo†*, Maria Ibáñez§*

Corresponding Authors:

* Maria Ibáñez: mibanez@ist.ac.at

https://ibanezgroup.pages.ist.ac.at

* Jonathan De Roo: Jonathan.deroo@unibas.ch

https://deroo.chemie.unibas.ch/en/

§ IST Austria, Am Campus 1, 3400 Klosterneuburg, Austria.

‡ Institute of Energy Technologies, Department of Chemical Engineering and Barcelona Research Center in Multiscale Science and Engineering, Universitat Politecnica de Catalunya, 08019 Barcelona, Spain

† Department of Chemistry, University of Basel, 4058 Basel, Switzerland.

Contents

Chemicals ... 3
Synthesis of CeO_2 NCs ... 3
Ligand stripping ... 3
Structural characterization of the CeO_2 NCs ... 4
Pair Distribution Function Analysis ... 5
X-ray photoelectron spectroscopy analysis ... 6
Determination of ligand coverage ... 7
Surface characterization ... 8
Structural elucidation of the products found in the reaction mixture ... 10
Identification of the secondary aldimine **6** ... 10
Hydrolysis of the secondary aldimine **6** ... 12
Synthesis of N-hexadecyldecylimine and spiking of the reaction mixture ... 13
Identification of hexadecanol **3** and spiking with oleyl alcohol ... 14
Identification of hexadecanenitrile **5** ... 14
Identification of the amide **9** ... 15
Identification of ammonia ... 16
Identification of the terminal alkene **2** ... 17
Temperature profile of the reaction ... 18
Synthesis with dioctadecylamine: Acid-free oxide particles from nitrates ... 20
Decomposition of other metal nitrates ... 21
References ... 23

Chemicals

Cerium (III) nitrate hexahydrate (99%), toluene (99.8%), oleic acid (OA, 90%), octadecane (99%), hexadecylamine (90%), oleylamine (OLA, 98% primary amine), dodecanenitrile (99%), decanal (>95%) and trifluoroacetic acid (99%) were purchased from Sigma Aldrich. Acetone (>99.5%) was purchased from Fischer Scientific. Chloroform-D (D, 99.8%) was obtained from Cambridge Isotope Laboratories. All chemicals were used as received except OLA which was vacuum distilled. All syntheses were carried out using standard airless techniques: a vacuum/dry nitrogen gas. An argon Schlenk line was used for nanocrystal syntheses and the particles were then washed in air unless otherwise specified.

Ammonia MQuant test stripes for semiquantitative Nessler determinations were obtained from Sigma Aldrich. Interference with OLA and dodecanenitrile was tested from saturated solution of the respective organics in water.

Synthesis of CeO_2 NCs

The syntheses of metal oxides were adapted from a reported synthesis of CeO_2.[1] In a typical synthesis $Ce(NO_3)_3{\cdot}6\ H_2O$ (434.8 mg, 1 mmol), 6 mmol of amine and 4mL of n-octadecane were mixed in a three neck flask. The mixture was degassed at room temperature and at 80°C for 30 minutes each. At 80°C a brown suspension forms. The flask is backfilled with argon and the temperature is increased to 300°C with a ramp of 15°C per minute. At 200°C the suspended solids dissolve completely, forming a clear solution and at about 240°C the reaction mixture turns dark brown. The reaction mixture is kept at 300°C for 60 minutes and afterwards it is cooled down in air until 160°C when 2 mL of toluene were injected. The mixture is precipitated with 25 ml acetone and centrifuged for 6 minutes at 6500rpm. For washing the particles, they are resuspended and precipitated with toluene (5 ml) and acetone (25 ml) and centrifuged 3 minutes at 3500 rpm. This washing procedure is repeated twice, and the particles were stored in 5 mL toluene.

Note: the reaction proceeds with strong gas evolution. It is therefore recommended to work with big flaks (for a standard synthesis 50 mL or bigger). In small flasks strong bubbling can cause the solution to rise though the condenser creating big temperatures differences in the reaction mixture.

Ligand stripping

For the characterization of bound ligands, NCs were stripped by adding 10 µL of pure trifluoroacetic acid to a solution of 50 mg of NCs in 0.5 mL $CDCl_3$. The particles precipitated, and the mixture was put in the ultrasonic bath for 30 min and subsequently dried under vacuum. $CDCl_3$ (0.6 mL) was added and after thorough mixing, the precipitate was filtered. The supernatant was measured in NMR.

Structural characterization of the CeO_2 NCs

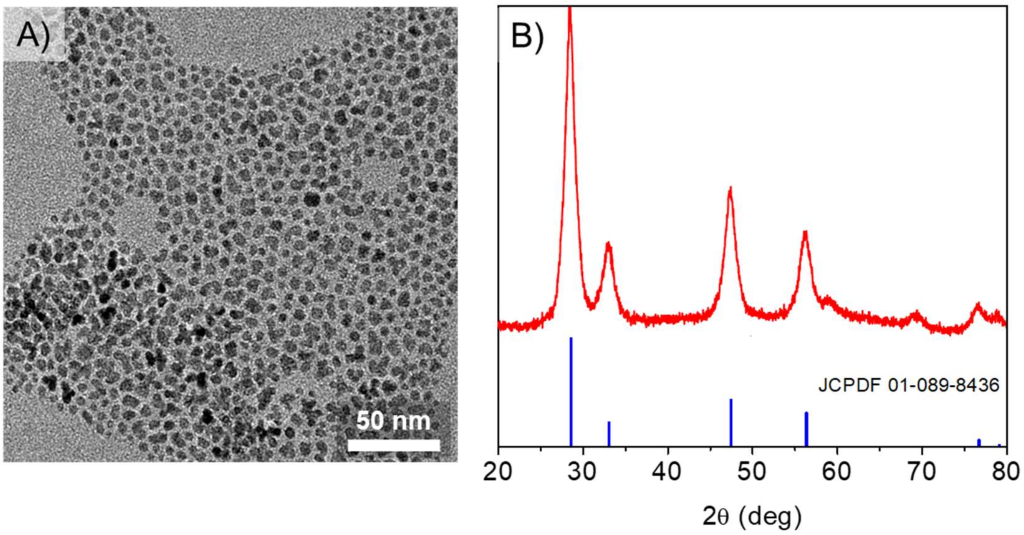

Figure S1: A) TEM micrographs of NCs synthetized with oleylamine in octadecane. B) Corresponding X-ray diffraction pattern of the NCs. A more detailed crystallographic analysis can be found in the section on Pair Distribution Function analysis, where we determine the average crystallite size (Figures S3 and S4).

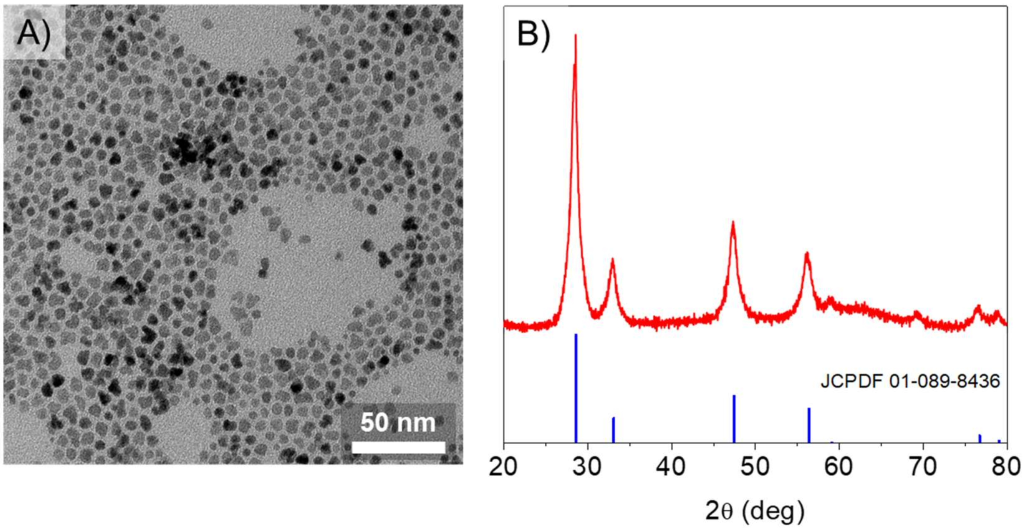

Figure S2: A) TEM micrographs of NCs synthetized with hexadecylamine in octadecane. B) Corresponding X-ray diffraction pattern of the NCs.

Pair Distribution Function Analysis

Total scattering X-ray PDF experiments were conducted at room temperature using Malvern Panalytical Empyrean Nano Edition lab source PDF diffractometer with Ag-Kα source (0.56 Å and 22.1keV). Samples were prepared in a 0.2 mm glass capillary. Data collection was carried out with 1D focusing X-ray mirror/slit system and a Galipix^{3D} hybrid pixel detector attached to 85mm radius reduction interface using Data collector software. The collected data was processed after proper background subtraction using Highscore Plus.[4] Q_{min} of 0.4 Å^{-1} and Q_{max} of 20.6 Å^{-1} was used to generate G(r). Diffpy-CMI was used for modeling and fitting.[5]

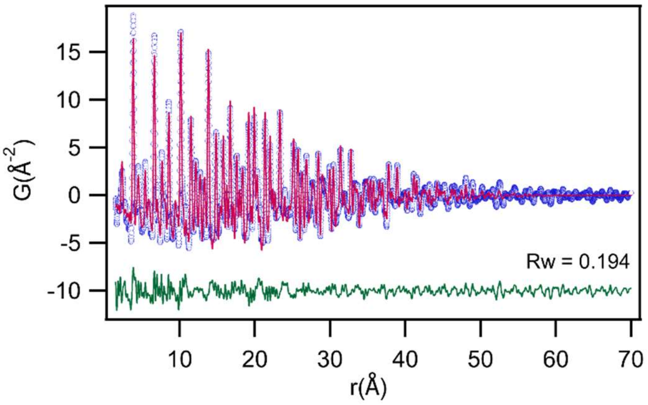

Rw	0.194
Lattice parameter, a	5.42 Å
Uiso_Ce	0.005 Å
Uiso_O	0.024 Å
psize	6.45 nm

Figure S3: X-ray PDF analysis of $CeO_{1.74}$. The figure shows the single-phase fit to the data, using the cubic crystal structure of ceria (Fm-3m). Due to the small x-ray scattering factor of oxygen, we cannot use PDF to refine the oxygen vacancies. Therefore, we use the oxygen occupancy (0.875) as derived from XPS.

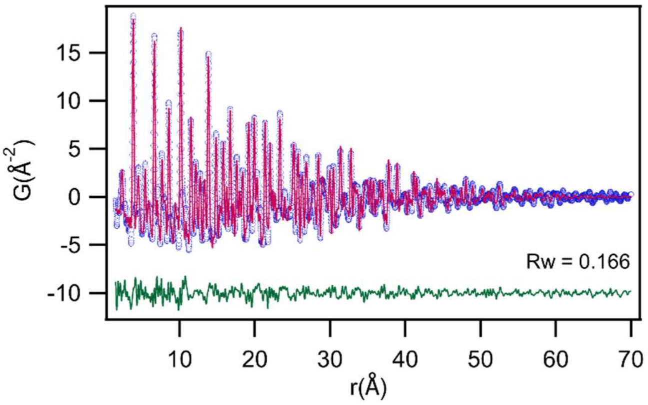

Rw	0.166
Lattice parameter, a	5.42 Å
Uiso_Ce	0.005 Å
Uiso_O	0.023 Å
Psize_1	7.86 nm
Psize_2	2.15 nm

Figure S4: X-ray PDF analysis of $CeO_{1.74}$. The figure shows the dual phase fit to the data, using the cubic crystal structure of ceria (Fm-3m). The fit is clearly improved (lower Rw) compared to the single-phase fit. We conclude the sample is quite polydisperse with a broad range of crystallite sizes (from 2 to 8 nm).

X-ray photoelectron spectroscopy analysis

We performed XPS measurements on different samples to determine the ratio $Ce^{(III)}/Ce^{(IV)}$ in the samples along with the presence of N, C and O. Since ceria gets reduced progressively under vacuum:

$$CeO_2(s) \rightarrow CeO_{2-x}(s) + \frac{x}{2}O_2(g)$$

We kept the samples similar times under vacuum (between 21 and 23 hs).

We analyzed particles prepared with hexadecylamine and particles prepared with dioctadecylamine. Moreover, we exposed the particles to air and analyzed them one week later to determine if the $Ce^{(III)}$ was oxidized changing the composition of the sample, and similar results were obtained.

Table S1: Atomic composition of the samples obtained from XPS

Ligands used/ sample treatment	C	N	O	Ce	$Ce^{(III)}/Ce^{(IV)}$	Formula
**Dioctadecylamine**	78.1	0.98	20.2	0.67	0.55	$CeO_{1.73}$
**Hexadecylamine**	61.2	0.34	36.8	1.67	0.53	$CeO_{1.74}$
**Hexadecylamine exposed to air**	58.2	0.59	39.1	2.12	0.52	$CeO_{1.74}$

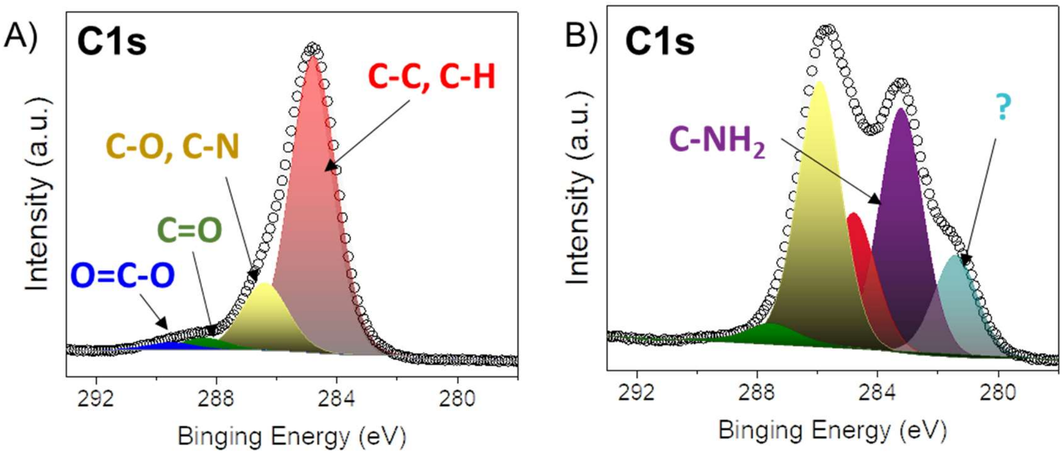

Figure S5: High resolution XPS spectra of the C1s region of A) particles synthesized with hexadecylamine and B) with dioctadecylamine. The C 1s peak of the sample with dioctadecylamine does not show any component at high binding energies, which are typical for the carboxylic group. Furthermore, the component labeled with a question mark has the typical shift of carbides and could not be identified.

Determination of ligand coverage

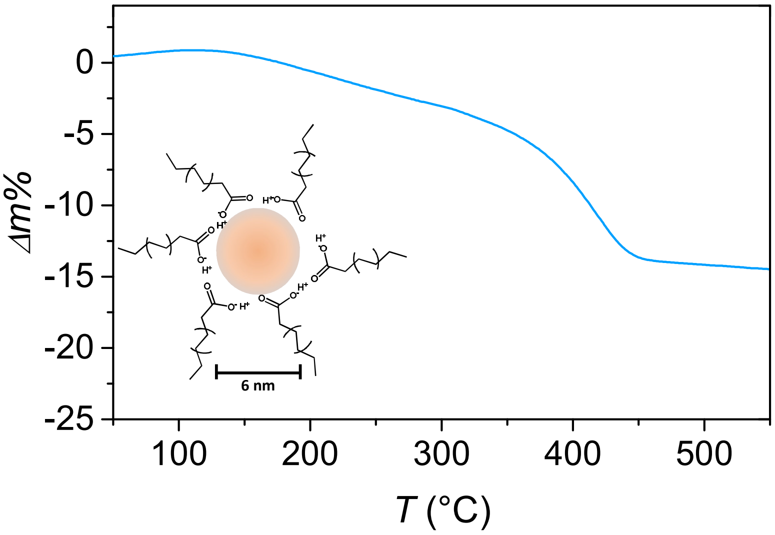

Figure S6: Thermogravimetric analysis (TGA) curve of nanocrystals prepared with hexadecylamine showing a total mass loss of ca. 15%. Measurements were performed in a TGA PT1000 (Linseis, Germany) from room temperature to 600 °C under a constant argon flow.

Calculation of ligand density:

Assuming that the mass loss in the TGA experiment corresponds to loss of hexadecanoic acid (palmitic acid, Mw = 256.43 g/mol), and using the average NC size from PDF (see further), 6.5 nm, a ligand coverage of 3.3 acid molecules per nm^2 can be calculated.

First the volume and surface area of a single nanocrystal are calculated.

$$V_{NC} = \frac{4}{3}\, \pi\, r^3 = \; 144\, nm^3$$

$$A_{NC} = 4\, \pi\, r^2 = 132\, nm^2$$

The mass of a single nanocrystal (without ligands) is calculated

$$m_{NC} = \delta_{the}\; .V_{NC} = 7.22\; g\; cm^{-3}\;\; 144\, nm^3 = \; 1.04\; 10^{-18} g$$

The mass of the ligand shell (of one nanocrystal) is calculated

$$m_{ligands} = \frac{0.15}{0.85}\, m_{NC} = 1.83\; 10^{-19} g$$

From the mass, the number of ligands is calculated

$$n_{ligands} = \frac{m_{ligands}}{M_w} N_a = 431\; molecules$$

And finally the ligand density is calculated

$$Ligand\; density = \frac{n_{ligands}}{A_{NC}} = 3.3\; nm^{-2}$$

Surface characterization

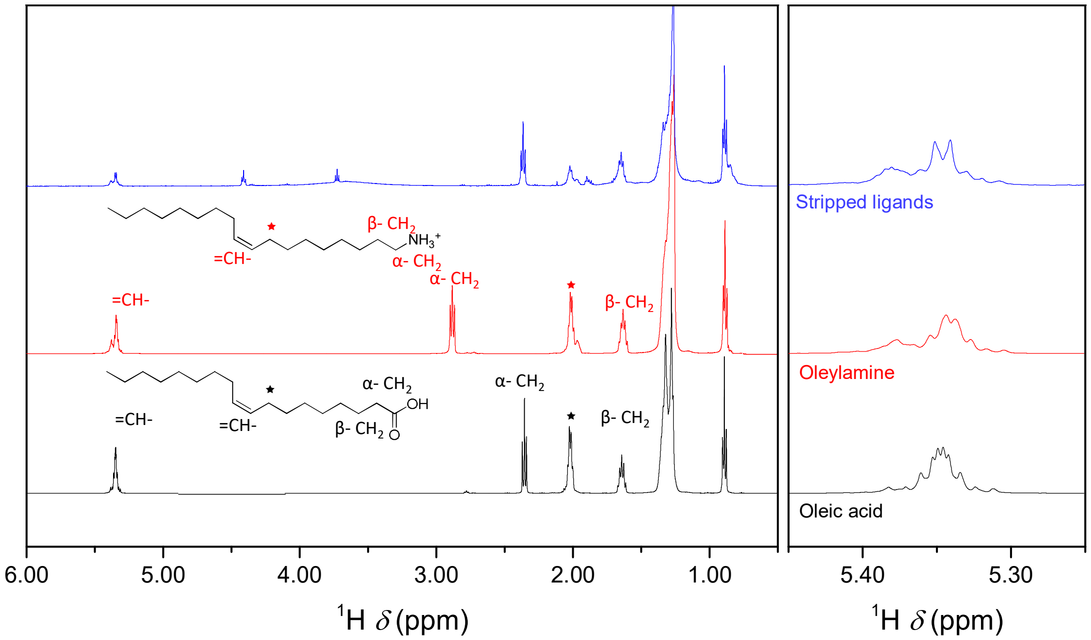

Figure S7: ^{1}H NMR spectra of CeO_{2-x} nanocrystals in prepared with oleylamine (green), ligands stripped from these nanocrystals with trifluoroacetic acid (blue), oleylamine protonated by trifluoroacetic acid (red) and oleic acid in the presence of trifluoroacetic acid (black). Alkene resonances of the ligands and references showing that the stripped ligands have the same mixture of cis and trans isomers than the oleylamine used.

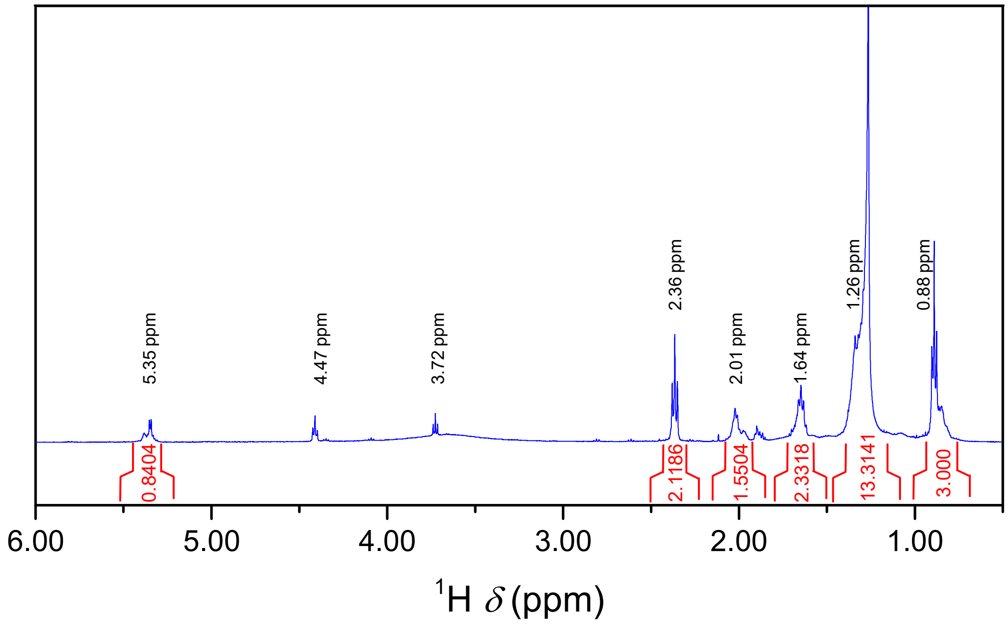

Figure S8: ^{1}H NMR spectrum of stripped ligands from CeO_{2-x} nanocrystals synthesized with oleylamine, with integrations.

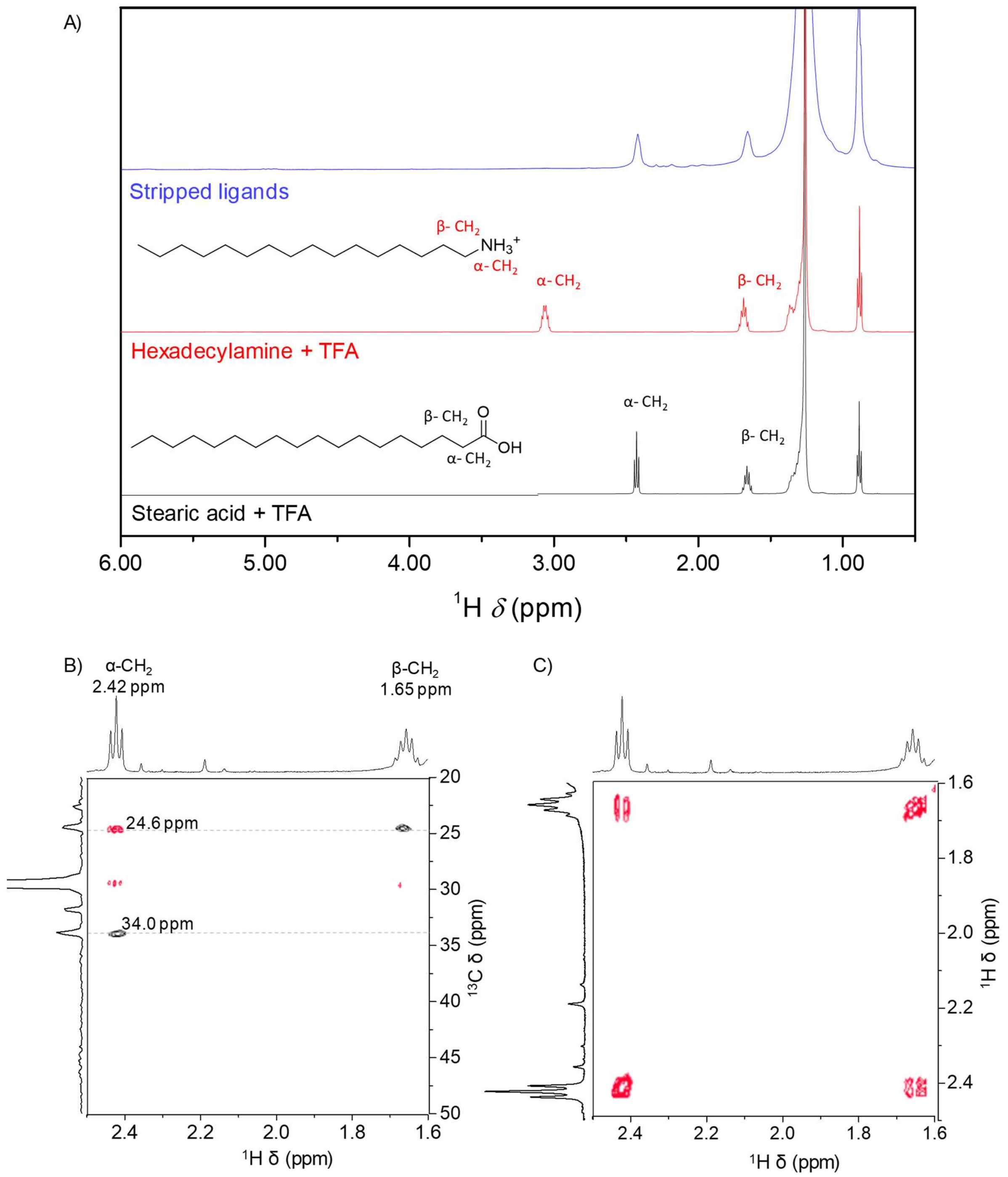

Figure S9: A) ^{1}H NMR spectra of ligands stripped from CeO_{2-X} nanocrystals in prepared with hexadecylamine (blue), protonated hexadecylamine (red) and protonated stearic acid (black) references. B) HSQC (black) and HMBC (red) spectra of the stripped ligand indicating the chemical shifts of the α and β carbon atoms, and C) COSY spectra of the stripped ligand.

Structural elucidation of the products found in the reaction mixture

Identification of the secondary aldimine 6

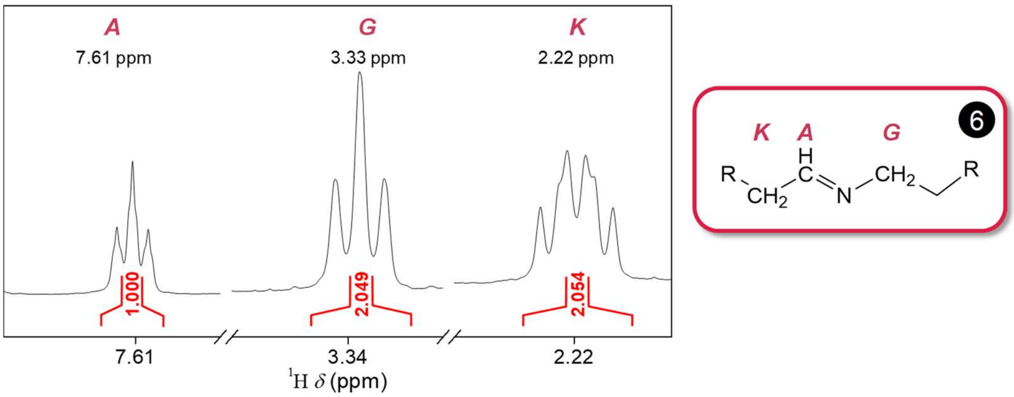

Figure S10: Magnification of the ^{1}H NMR resonances assigned to the aldimine **6**

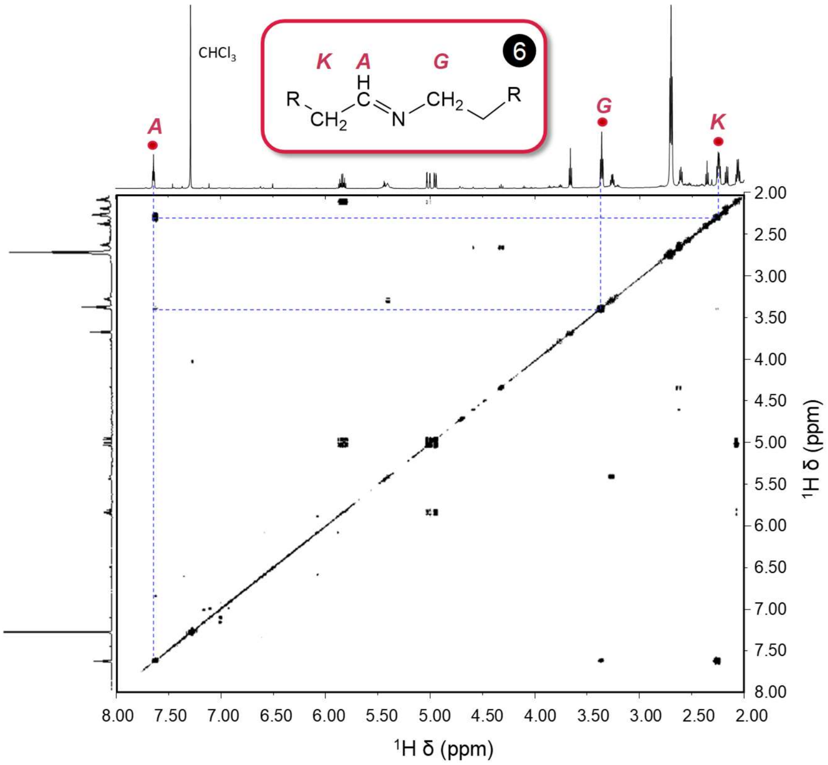

Figure S11: 2D COSY spectrum of the reaction mixture indicating the resonances assigned to the secondary aldimine **6**

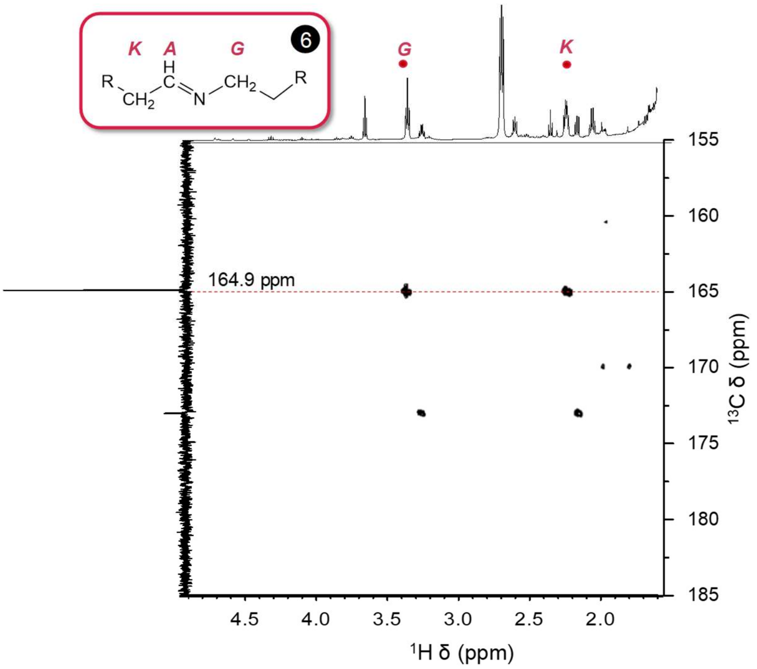

Figure S12: 2D HMBC spectrum of the reaction mixture indicating the correlation between the resonances *G* and *K* with the carbon of resonance *A*, assigned to the secondary aldimine **6**.

Hydrolysis of the secondary aldimine 6

To verify the identity of the aldimine **6** we hydrolyzed the reaction mixture. 200 µL of reaction mixture were mixed with a 400 µL H_2SO_4 (conc.) and 100 µL H_2O in a closed vial and heated for 90 minutes at 130 °C under strong stirring (>1000 rpm). The hydrolysed reaction mixture was extracted with 1 mL of ethyl acetate and dried under vacuum.

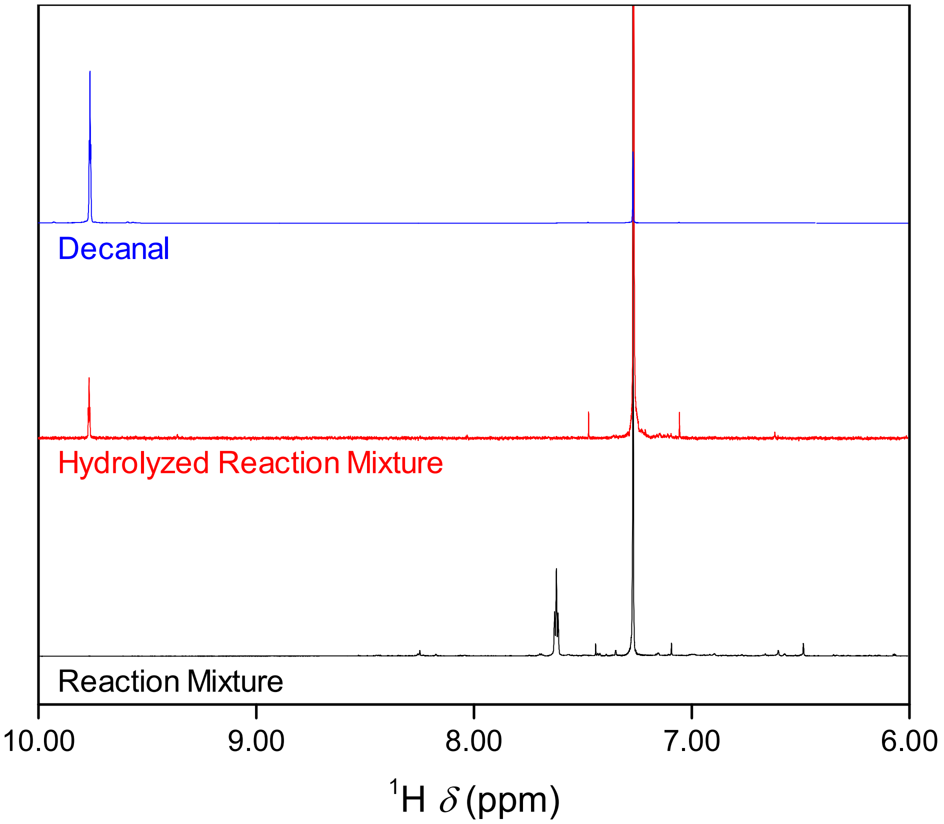

Figure S13: ^{1}H NMR spectra of the reaction mixture of a synthesis of CeO_{2-x} with hexadecylamine completion of a synthesis with hexadecylamine (black), hydrolyzed reaction mixture (red) and decanal (blue) as a reference. After hydrolysis the resonance *A* at 7.61 ppm (N_{imine}-H ; **6**) disappears and a new one at 9.76 ppm corresponding to the aldehyde **7** appears.

Synthesis of N-hexadecyldecylimine and spiking of the reaction mixture

We synthetized a N-hexadecyldecylimine to use as a reference. Briefly, decanal (190 µL, 1 mmol) and hexadecylamine (300,5 mg, 1.24 mmol) were mixed in round bottom flask connected to a vacuum line. The mixture was stirred under vacuum at 100 °C for 45 minutes to remove water formed during the condensation. Afterwards the reaction was cooled down in air and characterized by FTIR-ATR and NMR without further purification.

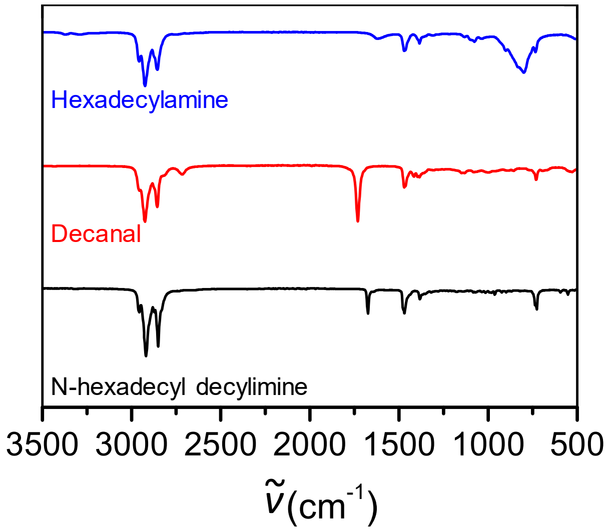

Figure S14: FTIR spectra of the reagents and products of the synthesis of N-hexadecyl decylimine. Hexadecylamine (blue), decanal red) and N-hexadecyldecylimine (black). The ν(C=O) stretching shifts from 1725 cm^{-1} (aldehyde) to 1668 cm^{-1} (imine) proving that the reaction took place.

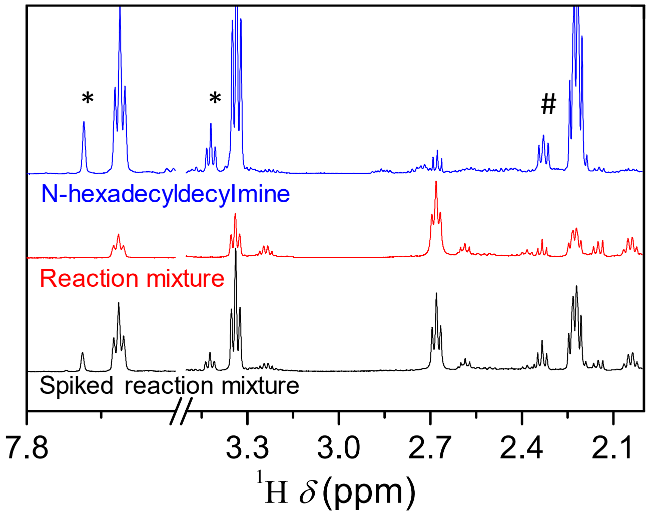

Figure S15: ^{1}H NMR spectra of the aldimine reference (blue), a reaction mixture (red), and the reaction mixture of a synthesis of CeO_{2-x} with hexadecylamine spiked with the aldimimine (black). The only change in the spectrum after spiking is the intensity of the peaks assigned to the aldimine, proving the identity of the compound. Residual peaks of the decanal (*) and hexadecylamine (#) are present in the aldimine.

Identification of hexadecanol 3 and spiking with oleyl alcohol

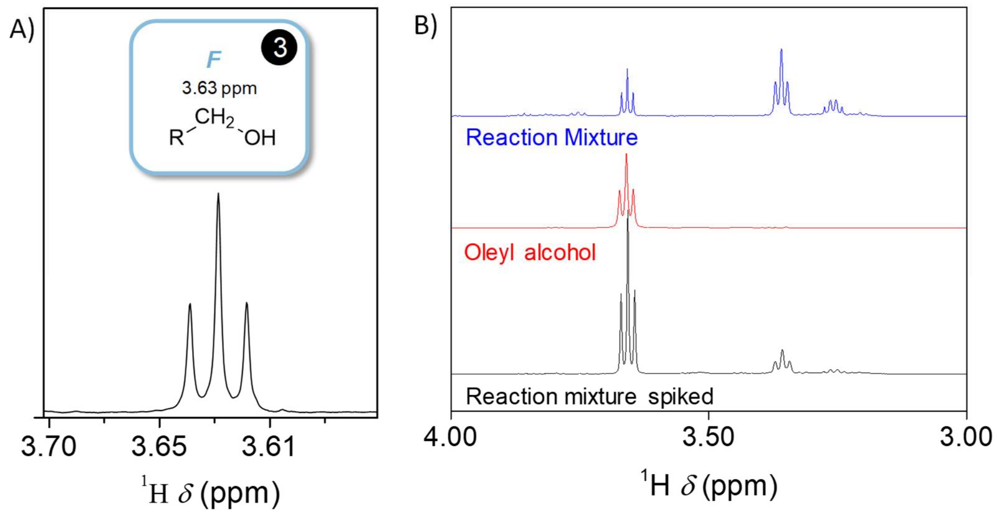

Figure S16: (A) Magnification of the ^{1}H NMR spectra of the reaction mixture showing the resonance assigned to the alcohol **3**. (B) ^{1}H NMR spectra of the reaction mixture (blue), oleyl alcohol (red), and the reaction mixture of a synthesis of CeO_{2-x} with hexadecylamine spiked with alcohol (black). The only change in the spectrum after spiking is the intensity of the peak assigned to the alcohol, proving the identity of the compound.

Identification of hexadecanenitrile 5

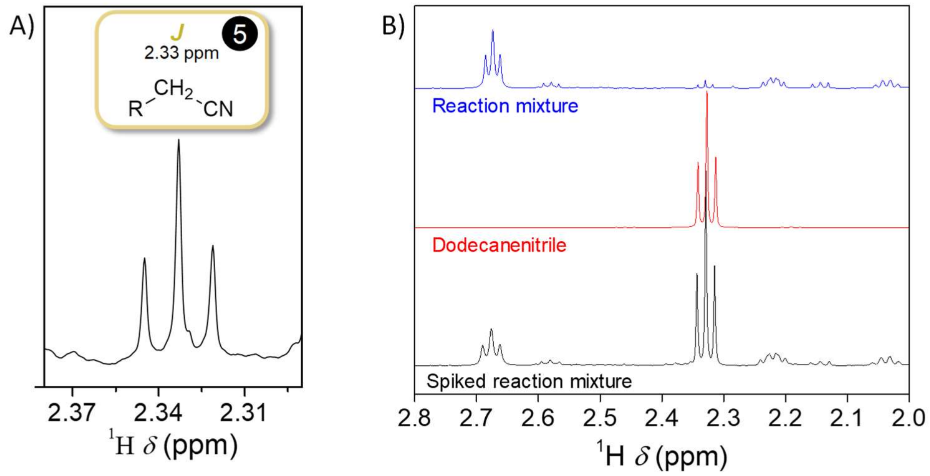

Figure S17: (A) ^{1}H NMR spectrum of the reaction mixture magnifying the resonance of the nitrile **6**; (B) ^{1}H NMR spectra of the reaction mixture of a synthesis of CeO_{2-x} with hexadecylamine spiked with dodecanenitrile (black), dodecanenitrile (red) and the reaction mixture (blue).

Identification of the amide 9

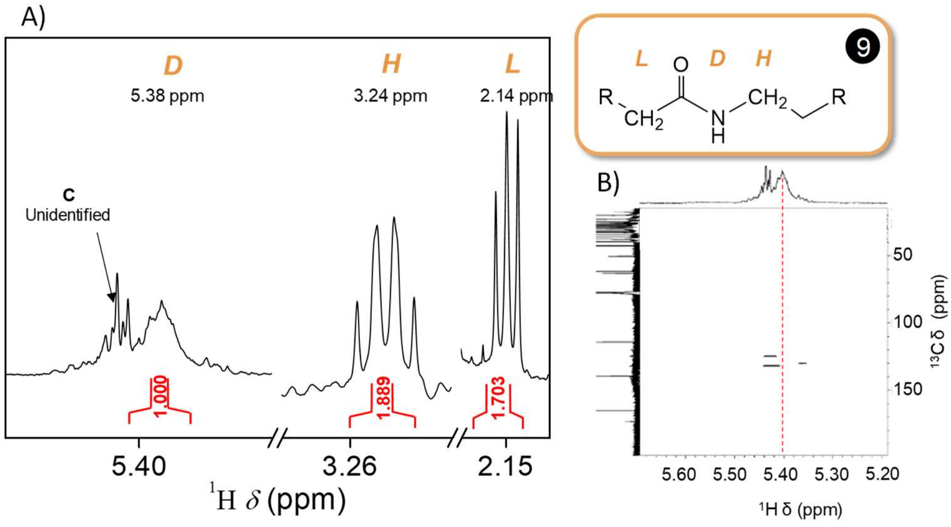

Figure S18: Magnification of the assigned ^{1}H NMR resonances assigned to the amide **9.** Resonance *D* overlaps with an unidentified peak, *C*. B) Magnified HSQC spectrum showing that, different from resonance *D*, the unidentified resonance *C* is bound to a carbon atom.

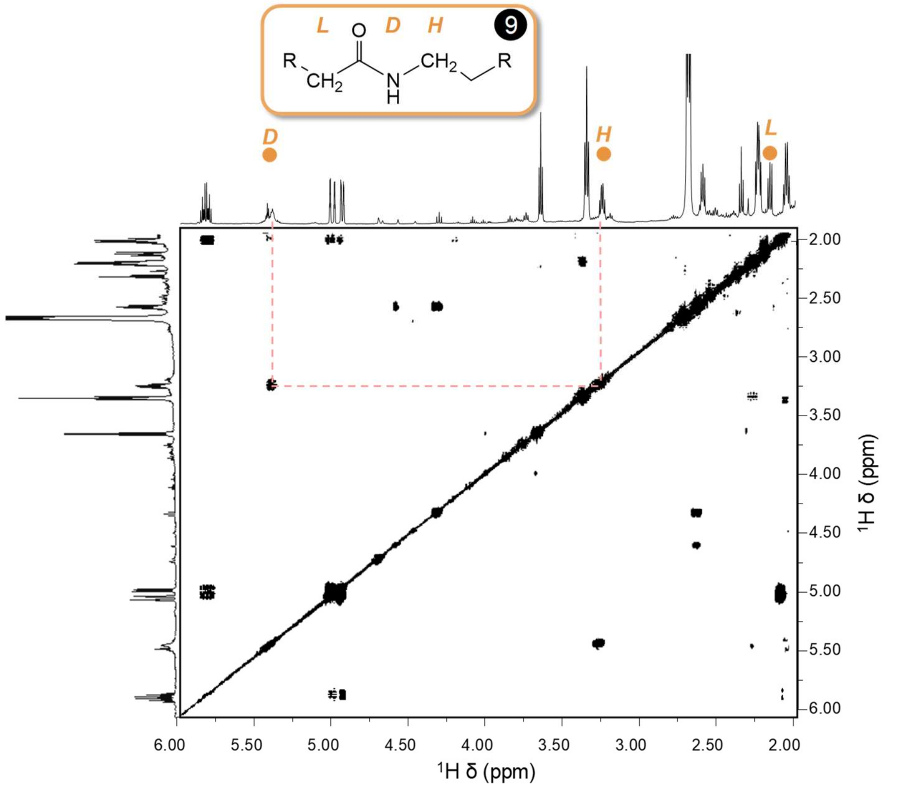

Figure S19: 2D COSY spectrum of the reaction mixture indicating the correlations between the resonances assigned to the amide **9**. Correlations with methylenes below 2 ppm are not included to avoid cluttering.

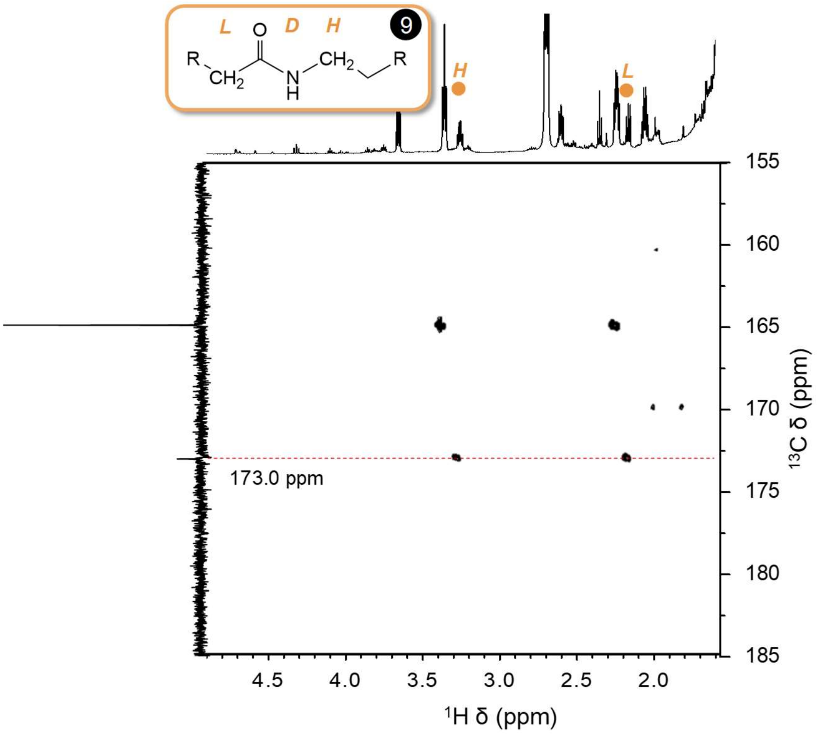

Figure S20: 2D HMBC spectrum of the reaction mixture indicating the correlation between the resonances *H* and *L* assigned to the amide **9**. The assignment is consistent with previous reports.[2]

Identification of ammonia

The gas mixtures in the headspace of the reactions (NiO, ZnO, ZrO_2 and CeO_2) was bubbled into water and the escaping gas still turns universal indicator paper blue, indicating the presence of a highly volatile basic gas in the mixture. The gas evolved during the heat-up of a normal synthesis was collected in acidulated water (pH = 2) and the pH was measured as the reaction proceeded rising steadily to 10.5.

Finally, to confirm that the evolved gas is ammonia we measured the concentration of ammonia in the aqueous solution with the Nessler method using commercial test stripes and found a concentration above 400 ppm. The test-kit showed no interference with neither the dodecanenitrile nor hexadecylamine, both scarcely soluble in water.

Identification of the terminal alkene 2

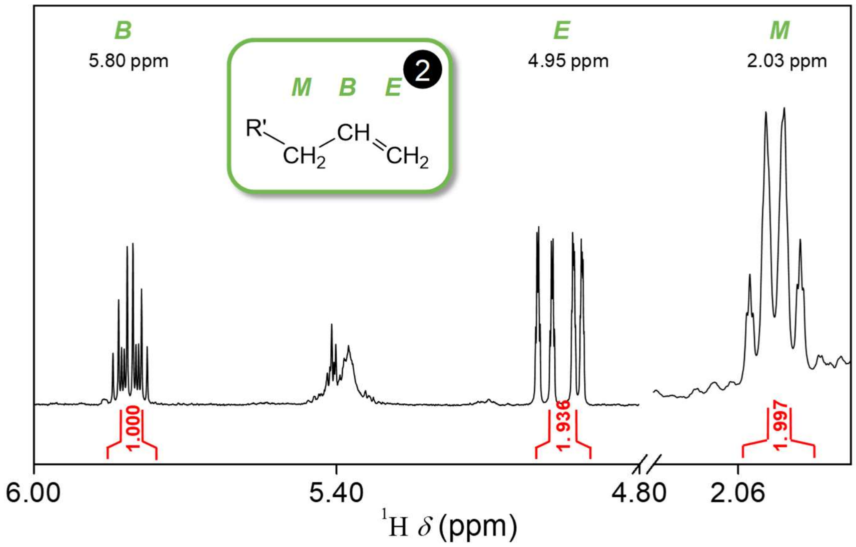

Figure S21: Magnification of the assigned ^{1}H NMR resonances assigned to the terminal alkene **2**.

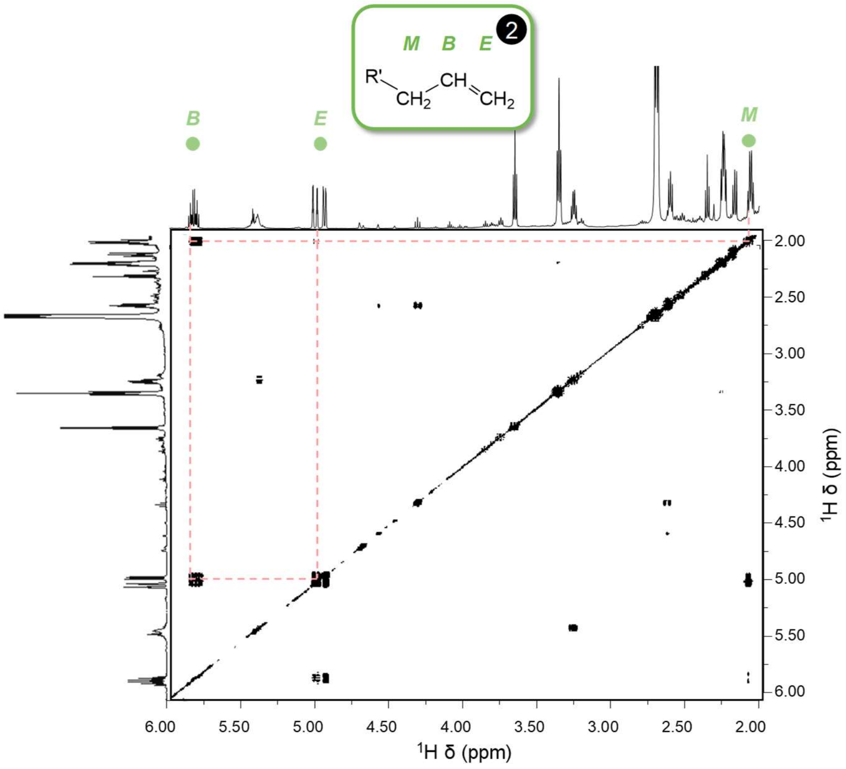

Figure S22: 2D COSY spectrum of the reaction mixture indicating the resonances assigned to the alkene **2**.

Temperature profile of the reaction

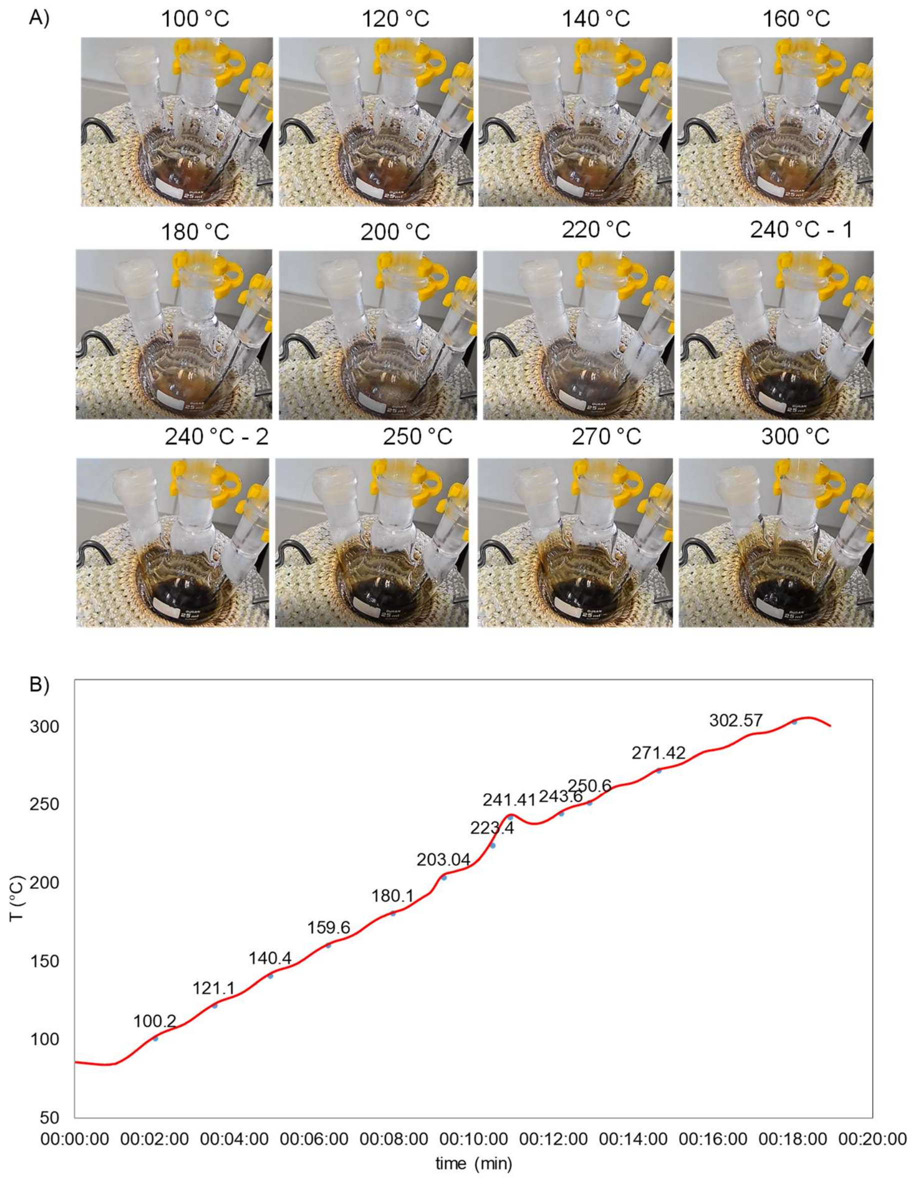

Figure S23: (A) Photographs of the reaction mixture of CeO_{2-x} taken during the heat-up to show the color change of the solution. The photos were acquired during aliquot extraction to have reliable data on the relationship between the reaction color, the size of the colloids and the reaction mixture. (B) Temperature profile of the reaction mixture for the aliquots taken in this section. The smooth profile shows that the extraction of aliquots does not affect the heating considerably. At 240°C the temperature remains constant for over 1 minute during which strong gas evolution is observed and the color of the reaction changes. This plateau in temperature corresponds to the endothermic decomposition of the metal nitrate.

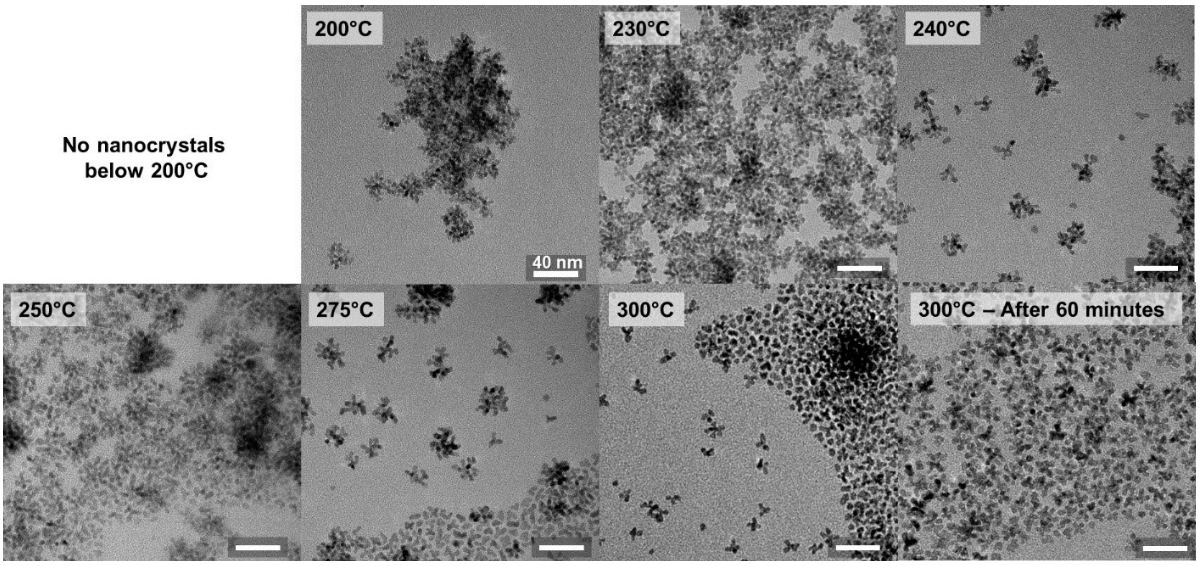

Figure S24: TEM micrographs of NCs found in aliquots taken at different times. The samples shown here are the same samples studied by NMR. No NCs were observed in aliquots taken below 200 °. The reaction mixture changes color upon heating up from a light brown suspension at low temperatures to a dark brown solution and stops changing color at 240 °C. The NCs continue to grow until 300 °C.

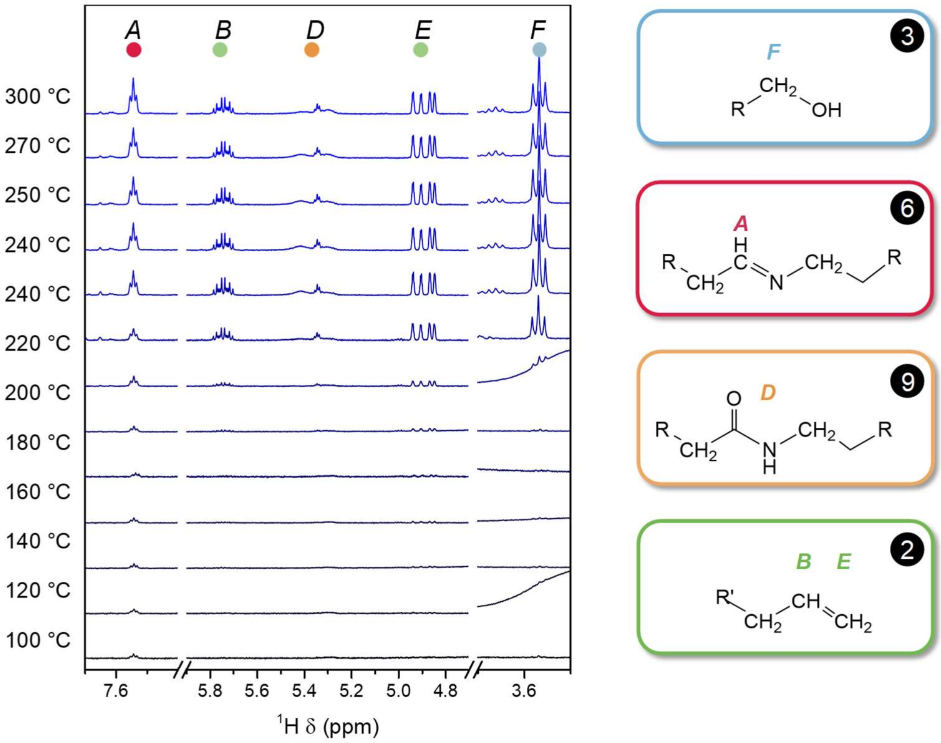

Figure S25: ^{1}H NMR spectra of aliquots of the reaction mixture taken at different temperatures during the heat-up. Broad resonances correspond to Ce(III) complexes.

Synthesis with dioctadecylamine: Acid-free oxide particles from nitrates

Acid free CeO_{2-x} particles were synthesized replacing oleylamine by dioctadecylamine in the procedure described in page S3 and using the same molar ratios. Given the low solubility of dioctadecylamine in polar solvents it coprecipitates with the nanocrystals in the purification procedure. Filtration and multiple (>10) purification steps are required to clean the nanocrystals.

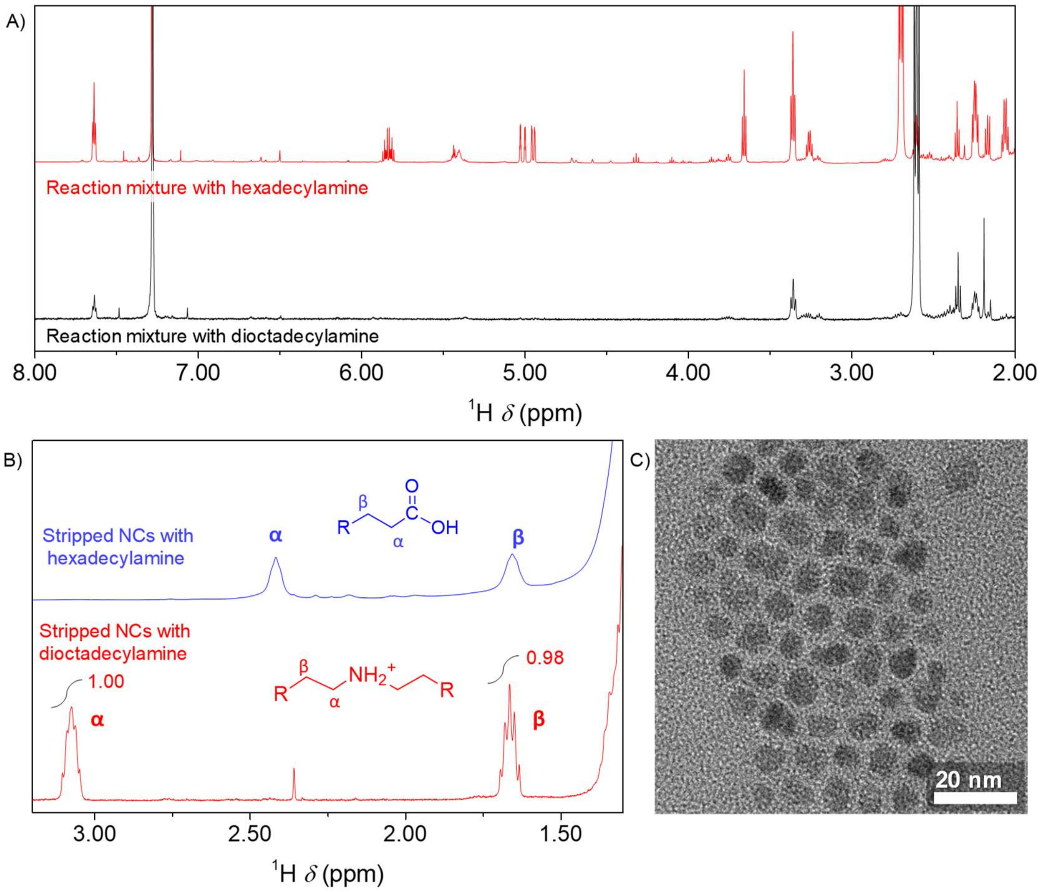

Figure S26: (A) ^{1}H NMR spectra of reaction mixtures of the synthesis of CeO_{2-x} with dioctadecylamine (black) and hexadecylamine (red). In contrast with the *normal* synthesis with hexadecylamine, the reaction mixture with dioctadecylamine shows no new resonances besides the formation of the aldimine **6**. (B) ^{1}H NMR spectra of ligands stripped from CeO_{2-x} NCs prepared with dioctadectylamine (red) and ligands stripped from NCs prepared with hexadecyalmine (blue) for comparison. (C) TEM micrographs of particles prepared with dioctadecylamine as capping ligand.

Table S2: Composition of the reaction mixture for different amines relative to the concentration of the starting amine (α-CH_2) based on the relative peak intensities

Ligand used for the synthesis	Aldimine 6	Alkene 2	Amide 9	Alcohol 3
**Hexadecylamine**	0.256	0.141	0.087	0.123
**N,N-dioctadecylamine**	0.133	0.027	0.007	0.027

Decomposition of other metal nitrates

The synthesis of other metal oxides was adapted from the synthesis of CeO_{2-x}. 1 mmol of metal nitrate hydrate was mixed with 6 mmol of hexadecylamine (1.45 g) in 4 ml of octadecane. The mixture was heated up to ~40 °C to melt the organic reagents before degassing under vacuum at 50°C and 100°C for 30 minutes each. After this time the nitrates solubilize completely. Afterwards the reaction mixtures were heated under argon up to evident color change (NiO: blue to green, ZnO: colorless to white) and finally cooled down in a water bath. Washing was done with several precipitation/redispersion steps using toluene as solvent and acetone as antisolvent. Centrifugation of the products in toluene was also done to separate the insoluble fraction (size selection).

A synthesis was also performed with zirconium (IV) oxynitrate hydrate, although it produced no colloidally stable nanocrystals, an insoluble ZrO_2 precipitate was recovered.

As a control experiment, we performed a heat up of $NaNO_3$ (1 mmol) with oleylamine (6 mmol) in 1-octadecene. No byproducts were observed in this reaction, although this might be attributed to the low solubility of $NaNO_3$.

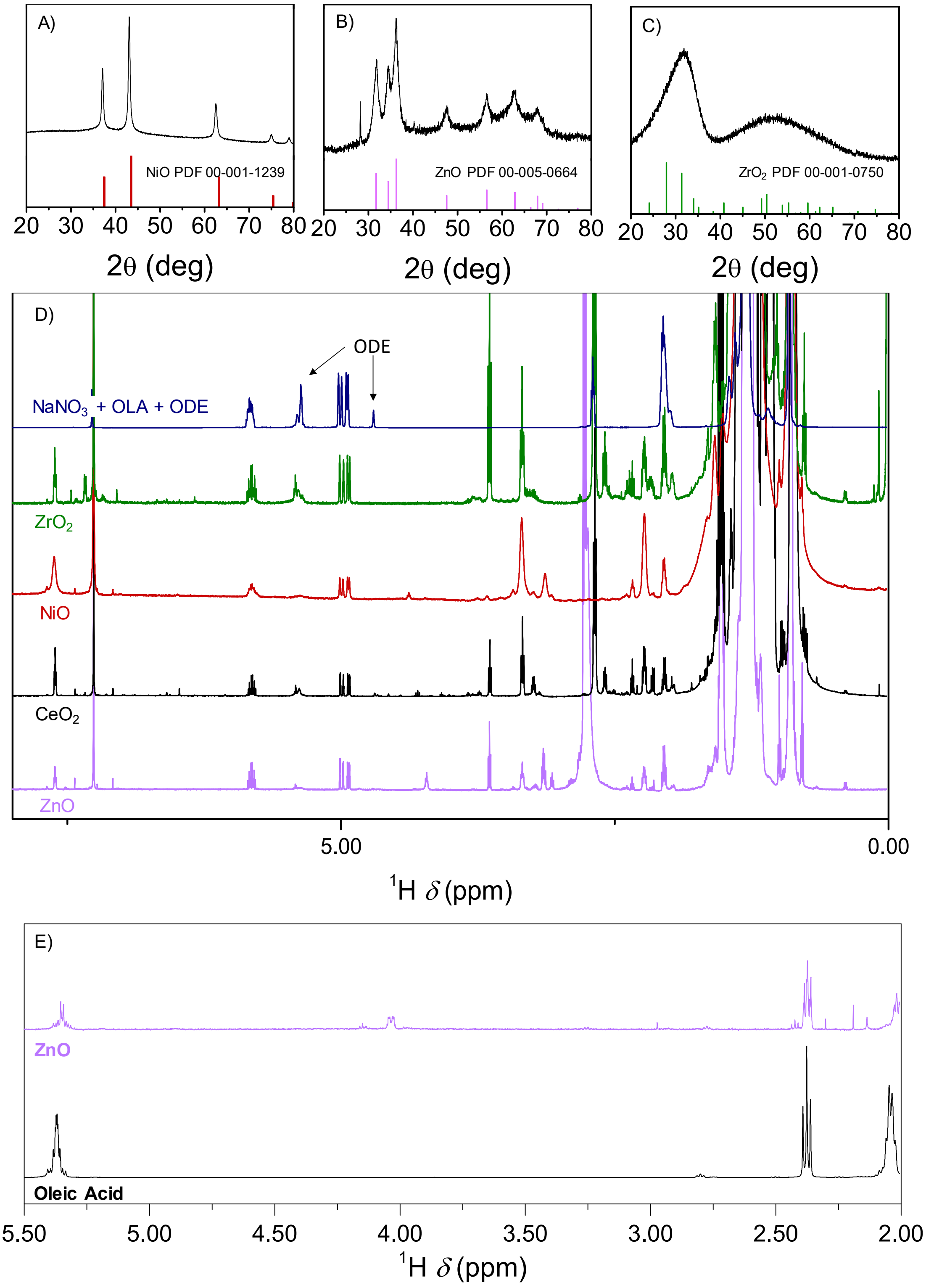

Figure S27: (A-C) XRD patterns of NiO, ZnO and ZrO_2 formed by the decomposition of the corresponding nitrates. (D) ^1H NMR spectra of the reaction mixtures with Zr, Ni, Ce and Zn nitrate precursors. (E) ^1H NMR spectra of ligands stripped from ZnO NCs together with an oleic acid reference spectrum.

References

(1) Berestok, T.; Guardia, P.; Blanco, J.; Nafria, R.; Torruella, P.; López-Conesa, L.; Estradé, S.; Ibáñez, M.; De Roo, J.; Luo, Z.; Cadavid, D.; Martins, J. C.; Kovalenko, M. V.; Peiró, F.; Cabot, A. Tuning Branching in Ceria Nanocrystals. *Chem. Mater.* **2017**, *29* (10), 4418–4424.

(2) De Roo, J.; Van Driessche, I.; Martins, J. C.; Hens, Z. Colloidal Metal Oxide Nanocrystal Catalysis by Sustained Chemically Driven Ligand Displacement. *Nat. Mater.* **2016**, *15* (5), 517–521.

(3) Clayden, J.; Greeves, N.; Warren, S. *Organic Chemistry*, 2nd ed.; OUP Oxford, 2012.

(4) Degen, T.; Sadki, M.; Bron, E.; König, U.; Nénert, G. The HighScore Suite. *Powder Diffr.* **2014**, *29* (S2), S13–S18.

(5) Juhás, P.; Farrow, C. L.; Yang, X.; Knox, K. R.; Billinge, S. J. L. Complex Modeling: A Strategy and Software Program for Combining Multiple Information Sources to Solve Ill Posed Structure and Nanostructure Inverse Problems. *Acta Crystallogr. Sect. A* **2015**, *71* (6), 562–568.
